# GSK3: A potential target and pending issues for treatment of Alzheimer's disease

**DOI:** 10.1111/cns.14818

**Published:** 2024-07-01

**Authors:** Jiahui Zhao, Mengying Wei, Minsong Guo, Mengyao Wang, Hongxia Niu, Tengfei Xu, Yuan Zhou

**Affiliations:** ^1^ School of Basic Medical Sciences Zhejiang Chinese Medical University Hangzhou China; ^2^ College of Pharmaceutical Sciences Zhejiang University Hangzhou China; ^3^ Future Health Laboratory, Innovation Center of Yangtze River Delta Zhejiang University Jiaxing China; ^4^ Cangnan County Qiushi Innovation Research Institute of Traditional Chinese Medicine Wenzhou China; ^5^ Key Laboratory of Blood‐stasis‐toxin Syndrome of Zhejiang Province Hangzhou China

**Keywords:** Alzheimer's disease, glycogen synthase kinase‐3, targeted drug, therapeutic target

## Abstract

Glycogen synthase kinase‐3 (GSK3), consisting of GSK3α and GSK3β subtypes, is a complex protein kinase that regulates numerous substrates. Research has observed increased GSK3 expression in the brains of Alzheimer's disease (AD) patients and models. AD is a neurodegenerative disorder with diverse pathogenesis and notable cognitive impairments, characterized by Aβ aggregation and excessive tau phosphorylation. This article provides an overview of GSK3's structure and regulation, extensively analyzing its relationship with AD factors. GSK3 overactivation disrupts neural growth, development, and function. It directly promotes tau phosphorylation, regulates amyloid precursor protein (APP) cleavage, leading to Aβ formation, and directly or indirectly triggers neuroinflammation and oxidative damage. We also summarize preclinical research highlighting the inhibition of GSK3 activity as a primary therapeutic approach for AD. Finally, pending issues like the lack of highly specific and affinity‐driven GSK3 inhibitors, are raised and expected to be addressed in future research. In conclusion, GSK3 represents a target in AD treatment, filled with hope, challenges, opportunities, and obstacles.

## Graphical abstract

GSK3 signaling in Alzheimer's disease and targeted therapies.

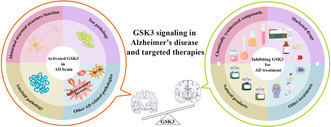

## 1 INTRODUCTION

**FIGURE 1:**
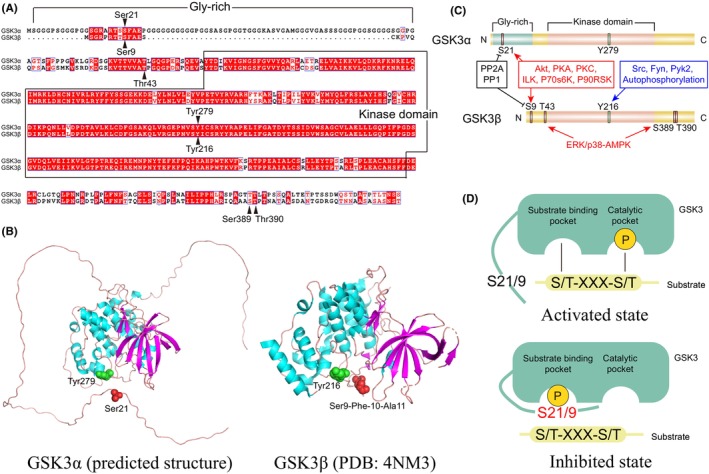
The structure and functional regulation of GSK3. (A) Amino acid sequence alignment of GSK3α and GSK3β. (B) Crystal structure of GSK3α (predicted) and GSK3β (PDB: 4NM3). The crucial phosphorylation sites, GSK3α (Ser21) and GSK3β (Ser9), are highlighted in red. GSK3α (Tyr279) and GSK3β (Tyr216) are marked in green. (C) Phosphorylation sites and upstream regulatory proteins of GSK3α and GSK3β. (D) The phosphorylation regulatory mechanism of GSK3. In the absence of phosphorylation at Ser21/9, GSK3 exists in an activated state. Its substrate binding pocket binds to substrates and facilitates the transfer of phosphate groups from the catalytic pocket. Conversely, when Ser21/9 gets phosphorylated, GSK3's substrate binding pocket tightly closes, inhibiting substrate binding and phosphorylation. Hence, GSK3 assumes an inhibited state.

Glycogen synthase kinase‐3 (GSK3) was first identified in the 1980s as a protein kinase responsible for phosphorylating and deactivating glycogen synthase in rabbit skeletal muscle.^1^, ^2^ Since then, GSK3 has been recognized as an evolutionarily conserved Ser/Thr protein kinase with a multitude of substrates. To date, over 100 substrates of GSK3 have been identified, with an additional 500 awaiting confirmation.^3^, ^4^ This extensive range of downstream regulated effects renders GSK3 one of the most functionally complex kinases involved in various cellular processes, including motility, metabolism, differentiation, proliferation, and apoptosis.^3^ The two isoforms of human GSK3, GSK3α, and GSK3β, consist of 483 amino acids (51 kDa) and 420 amino acids (47 kDa), respectively, as evidenced by research.^5^, ^6^ These isoforms are derived from chromosome 19 and chromosome 3, respectively.^6^, ^7^ Both GSK3α and GSK3β have been detected in almost all species, with over 90% similarity across different species.^8^ In Homo sapiens, the amino acid sequences of GSK3α and GSK3β show 84%–85% similarity, while their kinase domains exhibit a high degree of homology, with 19 differential amino acids from the 285 amino acids that make up the kinase domains (Figure 1A), according to sequence alignment.^6^, ^7^ Though hitherto no accurate structural information of GSK3α is obtained. Predicted result in UniProt (https://www.uniprot.org/uniprot/P49840#structure) suggests that the glycine‐rich region distinguish between these two isoforms does not form a stable secondary structure. Hence, the secondary structures of GSK3α and GSK3β (Figure 1B) are also thought to be similar especially in kinase domains, which arrive at a conclusion that GSK3α and GSK3β showed approximate biological activity. Nonetheless, research confirms that the glycine‐rich region plays a role in localizing GSK3α inside the cell.^9^ The wild‐type GSK3α is typically situated in the cytoplasm rather than the nucleus, as stated in scholarly literature.^7^ Intriguingly, research indicates that truncated GSK3α lacking the N‐terminal region congregates in the nucleus, with calcium stimulation further amplifying this effect.^10^ In contrast, GSK3β displays greater nuclear mobility, particularly during the S‐phase of the cell cycle and in the context of apoptosis, as reported in studies.^11^, ^12^

Alzheimer's disease (AD) is a neurological condition that is typified by cognitive impairment and memory loss. It is the foremost cause of dementia, and while it remains a growing concern, the ever‐increasing number of patients coupled with slow drug development has resulted in AD becoming a condition that is among the most destructive, expensive, and burdensome.^13^, ^14^ For instance, in the United States alone, it has been estimated that the cost of AD was approximately $305 billion in 2020, with around 5.8 million Americans affected.^15^ In view of latest statistics, it can be anticipated that the figure of affected individuals is likely to rise to 13.8 million by 2050.^15^ While acetylcholinesterase inhibitors like donepezil and excitatory amino acid receptor antagonists such as memantine remain the preferred treatment options for AD, it is worth noting that they have been approved by the U.S. Food and Drug Administration (FDA) for multiple decades and offer only partial relief of symptoms.^16^, ^17^ Recently, another drug called Aduhelm (aducanumab), which is a monoclonal antibody that targets Aβ (amyloid beta), has received FDA approval for treating AD.^18^ However, there is still ongoing debate regarding its curative effectiveness.^19^ Despite exhaustive research, there exists an incomplete understanding of the fundamental mechanisms at play in AD. Supported by characteristic pathological markers including the aggregation of Aβ and the formation of neurofibrillary tangles (NFTs), these factors are believed to be the primary contributors to AD.^13^ Furthermore, observations of inflammation,^20^ mitochondrial malfunction, oxidative stress,^21^ autophagy impairment,^22^ gut microbiota deregulation^23^ and occurrences of ferroptosis^24^ have all been documented in relation to the onset and progression of AD. Above all, AD is a highly intricate neurodegenerative disorder that requires ongoing exploration of therapeutic targets and drug development efforts.^13^, ^14^

High expression of GSK3 in the brain stands out as a significant phenomenon compared to its expression in other tissues.^6^ This finding was first noticed in animal models at both mRNA and protein levels and subsequently mirrored in the human brain.^25^, ^26^ Given its considerable expression levels, the role of GSK3 in normal brain physiology and neurological conditions such as bipolar disorder, AD, Parkinson's disease, and psychiatric disorders has become increasingly intriguing to researchers.^7^ Taking into account the multifunctional nature of GSK3 and its relevance to several pathological features of AD, notably the formation of NFTs,^27^, ^28^ significant attention has been devoted to GSK3 in the course of developing drugs for AD.^29^ The purpose of this paper is to comprehensively explicate the link between GSK3 signaling and AD, and to summarize the latest advances in drug development for AD that specifically target GSK3. Ultimately, the intention is to supply relevant guidance on basic research on GSK3 signaling and to chart potential directions for creating AD drugs that specifically target GSK3.

## 2 THE FUNCTION AND REGULATION MECHANISM OF GSK3

### 2.1 The location and function of GSK3 in the brain

From a spatial perspective, the brain is the organ with the highest abundance of GSK3 in healthy adults.^6^ However, from a temporal perspective, the content of GSK3 in the brain of mature rats is significantly higher than that of fetal rats and newborn rats.^30^ This phenomenon is related to the important role of GSK3 in brain development, while in the mature brain, GSK3 may primarily play a role as a maintainer of function. Research involving the transfection of primary neurons with shGSK3 has demonstrated significant axon growth inhibition.^31^ In cases where GSK3 is specifically eliminated in astrocytes, mice display excessive anxiety and anomalous social behaviors.^32^ Additionally, neural progenitor cells from GSK3 KO mice are prone to massive proliferation instead of differentiating into intermediate neural progenitors and postmitotic neurons.^33^ Meanwhile, cortical progenitor cells from GSK3 KO mice exhibit a disruption of radial migration and dendritic orientation.^34^

Noteworthily, GSK3α and GSK3β do not exhibit complete functional equivalence. GSK3α knockout (KO) mice, for example, are able to survive but have a shortened lifespan and male infertility.^35^, ^36^, ^37^ These mice also experience considerable abnormalities in cerebellum structure and demonstrate various mental disorders and behavioral deficits, including increased emotionality, reduced depression‐associated behaviors, decreased social interaction and aggression, altered information processing, and impaired long‐term memory function.^38^ In addition, GSK3α KO mice exhibit enhancements in glucose and insulin sensitivity, along with a reduction in fat mass.^35^, ^38^

**FIGURE 2:**
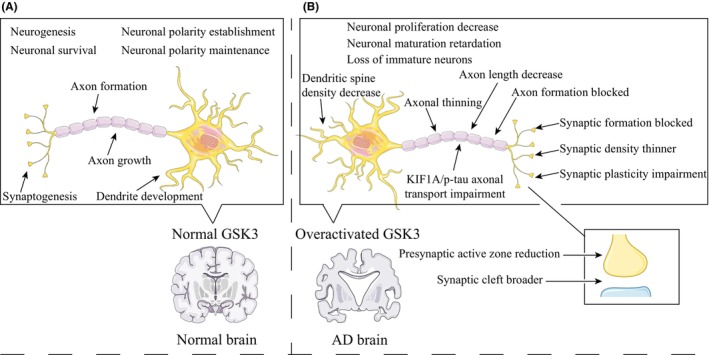
The role of GSK3 in neuronal growth, development, and function. (A) The conventional levels of GSK3 in normal brain and its positive impact on neurons. (B) The detrimental effects of GSK3 hyperactivation in the brains of AD patients on neuronal structure and function.

The GSK3β gene undergoes alternative splicing, resulting in the production of a neuron‐specific long form called GSK3β2.^39^ This isoform is enriched during brain development,^40^, ^41^ and plays a significant role (Figure 2A) in promoting neurodevelopment such as neurogenesis,^42^, ^43^ axon formation and growth,^30^, ^31^, ^44^ synaptogenesis,^45^ dendrite development, and neuronal survival.^28^ Furthermore, GSK3β is crucial for both the formation and maintenance of neuronal polarity (Figure 2A).^46^ Mice with complete KO of the GSK3β gene exhibit evident structural and functional abnormalities in the brain, but they succumb to liver and heart dysfunction in the early embryonic stage.^47^, ^48^ Heterozygous mice with GSK3β KO can survive into adulthood, yet they display reduced exploratory behavior,^49^ decreased aggression,^50^ and impaired memory.^51^ Furthermore, silencing GSK3β solely in the dentate gyrus of the hippocampus leads to an antidepressant‐like state in mice.^49^, ^52^, ^53^

There are also differences in the spatial distribution of GSK3β and GSK3α in the brain. The Allen Brain Atlas shows that GSK3β is expressed relatively evenly throughout the brain regions,^25^, ^54^ while GSK3α is only significantly expressed in the adult brain cortex, hippocampus, striatum, and cerebellum.^6^ To sum up, although GSK3 plays a crucial role in the generation and development of the brain, the levels of GSK3α/GSK3β are tightly regulated both spatially and temporally. The distinct distribution of GSK3α and GSK3β in the brain is likely associated with their functional differences. An experiment using mutant mice with GSK3α and GSK3β mutations demonstrated that GSK3α plays a more critical role than GSK3β in hippocampal bidirectional synaptic plasticity.^55^ The dysregulation of GSK3 is indeed a key contributing factor to various neurological disorders, making the maintenance or restoration of GSK3 homeostasis an important subject of research in the field of neurological diseases.

### 2.2 Regulation of the GSK3 activity

As a protein kinase, GSK3 possesses the ability to phosphorylate almost all downstream proteins that bear the S/T‐X‐X‐X‐S/T(P) motif, as evidenced by numerous substrates.^4^, ^56^ To date, crystal structures of GSK3β have been acquired, mainly due to its shorter sequence. These structures serve as the foundation for GSK3β's phosphate‐primed substrate specificity and autoinhibition.^57^ It is anticipated that GSK3α operates in a comparable manner.^4^ As shown in Figure 1D, the specific motif leads to a functional conformational change in GSK3, allowing substrate binding and phosphorylation.^4^, ^58^, ^59^ A groove adjacent to the catalytic pocket plays a crucial role in recognizing and accommodating S/T from the specific motif, and is therefore named the substrate binding pocket.^57^, ^58^ It is important to note that the groove discussed is significant for two exact opposite states of GSK3β, namely p‐GSK3β^Ser9^ and p‐GSK3β^Tyr216^. This is illustrated in Figure 1D, where Ser9 phosphorylation results in an inhibited state of GSK3β by altering the position of the loop and occupying the substrate binding pocket with the phosphorylated Ser9, thereby preventing GSK3β and substrates from binding.^60^ This phosphorylation state is crucial for maintaining normal cell homeostasis.^61^ In contrast, phosphorylation of Tyr216 activates GSK3β as the side chain of Tyr216 occupies the substrate binding pocket prior to phosphorylation and releases it after phosphorylation.^58^, ^62^ The states of p‐GSK3α^Ser21^ and p‐GSK3α^Tyr279^ reflect the inhibited and activated state of GSK3α, respectively, and although no direct evidence from structural biology is available, the identical inhibited and activated state suggests a similar phosphorylation regulatory mechanism for these two isoenzymes.^62^, ^63^

Not only catalyzing many substrates, GSK3 is under dynamical regulation of various of kinases as well (Figure 1C). Ser21/9 can be phosphorylated by protein kinase B (PKB), commonly known as AKT, as well as its highly homologous proteins protein kinase A (PKA) and protein kinase C (PKC), along with Integrin‐linked kinase (ILK), p70S6K, and p90RSK.^7^, ^64^ Additionally, two subfamilies of mitogen‐activated protein kinase (MAPK), extracellular signal‐regulated kinases (ERK) and p38, play a role in the phosphorylation of Thr43, Ser389, and Thr390, which results in GSK3β Ser9 phosphorylation.^65^, ^66^ The activating kinases of GSK3β have been previously documented to perform the crucial function of phosphorylating GSK3β at Tyr216. Specifically, Src, Fyn, and Pyk2 have been identified as the kinases responsible for this activation process.^7^ In addition, it should be highlighted that GSK3β is also capable of performing autophosphorylation at Tyr216.^67^ Of note, Ser21/9 dephosphorylation is also reported for GSK3 activity regulation, protein phosphatase 2A (PP2A) and protein phosphatase 1 (PP1) have been identified as the key enzymes involved in this process.^68^

## 3 THE ROLE OF GSK3 IN AD

There is a wealth of evidence that supports a strong correlation between GSK3 and AD. Studies have shown that inhibiting the state of GSK3β, specifically p‐GSK3β^Ser9^, is beneficial for long‐term memory formation.^69^ Through the identification of genes and cerebrospinal fluid biomarkers in hundreds of AD patients and healthy volunteers, it has been revealed that genetic variants of GSK3β are closely associated with Aβ and p‐tau.^70^ Moreover, high activity of GSK3β in the peripheral blood of AD patients has been found to be positively correlated with the severity of dementia.^71^ Animal models have provided similar insights, as both the content and activity of GSK3β were observed to increase with age.^72^, ^73^, ^74^ In a CamKIIα‐tTA/GSK3β mouse model, where GSK3β was overexpressed starting at 6 months, neurodegeneration and other AD symptoms were observed at 12 months of age.^75^ Further mechanistic studies have revealed that the excessive activity of GSK3 influences the onset and progression of AD through various pathways.

### 3.1 Impairment of the neuronal structure and function

Given the complex biological functions of GSK3 and its precise regulation, the dysregulation of GSK3, especially the excessive activation in AD patients or animal models, can have multiple negative effects on neurons (Figure 2B). Researchers have activated GSK3 in model animals using different methods, and although the focus and specific indicators observed may vary, they have all discovered similar abnormalities in neuronal structural and functional processes. Abnormal neuronal proliferation and maturation processes, as well as resulting dendritic atrophy, have been found in CamKIIα‐tTA/GSK3β mice.^76^ Similarly, rats carrying the GSK3β mutant with Ser9 mutated to Ala experience sustained activation of GSK3β, leading to significant disruptions in neuronal oscillations in the prefrontal cortex and hippocampal ventral side.^77^ Conversely, pharmacological inhibition of GSK3β can promote neurogenesis in APP/PS1 mice.^78^

Specifically, GSK3 overexpression or excessive activation also damages substructures within neurons, such as synapses, dendrites, and axons. GSK3β overexpression can inhibit synaptogenesis.^79^ In rats with elevated GSK levels caused by exogenous or endogenous factors, the presynaptic active zone, postsynaptic density, and synaptic cleft are all negatively affected, accompanied by long‐term potentiation (LTP) inhibition.^80^ LTP inhibition and related synaptic damage are direct causes of memory impairment.^81^, ^82^, ^83^ Excessive activation of GSK3β in STZ‐induced sporadic AD rats ultimately leads to decreased dendritic spine density and axonal thinning in the hippocampal CA1 region.^84^ In AD mouse models exposed to Aβ oligomers, excessive GSK3β activation is accompanied by dendritic spine loss.^85^ Axonal transport function is also severely impaired by GSK3 overactivation, leading to excessive accumulation of p‐tau in the distal axon and impaired axonal transport of KIF1A (an essential protein for the delivery of neurotrophic factors), both contributing to the development of AD.^86^, ^87^, ^88^, ^89^

In terms of mechanisms, based on the existing reports, the interaction between presenilin 1 (PS1) and GSK3 appears to be a crucial factor. PS1 serves as a significant biomarker of AD and has been demonstrated to be associated with the incidence of the disease, being utilized for establishing AD animal models through genetic mutations.^90^ Phosphorylation of PS1 by GSK3β weakens its interaction with N‐cadherin/β‐catenin, resulting in the formation of trimeric complexes at synapses and a decrease in synaptic plasticity and neuronal vitality.^91^ Mice with PS1 deficiency or mutations display increased GSK3β activity, leading to notable axonal transport impairments. Mechanistically, GSK3β is involved in the augmented release of membrane‐bound organelles driven by kinesin‐I.^92^ Furthermore, excessive activation of GSK3β increases the phosphorylation level of collapsin response mediator protein 2 (CRMP2), which hinders CRMP2 signal transduction and its regulation of axonogenesis and synaptic formation.^93^, ^94^

GSK3 is a critical participant in the β‐catenin‐dependent Wnt signaling pathway.^95^ The pathway is known to have a significant impact on synaptic plasticity as well as memory.^96^ The Wnt family is comprised of 19 lipid‐modified glycoproteins and plays a crucial role in a range of cellular processes including cell metabolism, cell fate determination, polarity, and cytoskeleton alterations. The activation of the β‐catenin‐dependent Wnt signaling pathway involves the binding of Wnt to various cell surface receptors such as Frizzled (Fz), low‐density lipoprotein receptor‐related protein 5 (LRP5), and LRP6, ultimately triggering β‐catenin to exert its biological effects.^95^ GSK3 is a vital regulator of β‐catenin degradation, and it forms a complex with casein kinase Iα (CKIα), Axin, and adenomatosis polyposis coli (APC) to phosphorylate β‐catenin. Subsequently, E3 polyubiquitin ligases recognize the phosphorylated β‐catenin, and it is degraded by proteasome. Upon activation of Wnt signaling, the complex is eliminated, inhibition of GSK3 stabilized β‐catenin, thus as a transcriptional coactivator that translocates to the nucleus to promote transcription of related genes.^95^ Wnt‐dependent protein degradation is a common occurrence in cells when GSK3 dysfunction manifests. GSK3's involvement in the Wnt/β‐catenin pathway aggravates AD‐associated neuron damage.^97^

Following an evaluation of gene expression in the prefrontal cortex of both normal brains and AD patients, it was observed that the protein levels and activities of β‐catenin and GSK3β underwent a dynamic switch. These proteins are associated with Wnt signaling and suggest that such signaling is significantly impaired in AD patients.^98^ Further research has indicated that overexpression of the Wnt antagonist Dickkopf‐1 (Dkk‐1) in the hippocampus can lead to a reduction in learning and memory ability in mice. This effect was linked to synapse loss, LTP impairment, and long‐term depression enhancement. However, the negative consequences were eliminated through associative inhibition of GSK3 and RhoA‐Rock. Repression of Dkk‐1 expression displayed a similar effect.^99^ Overall, GSK3β plays a negative role in Wnt signaling‐based neural functional recovery. Notably, activating muscarinic receptors inhibited GSK3β and restarted the Wnt/β‐catenin pathway in Aβ_1‐40_‐managed hippocampal neurons and glutamate‐managed PC12 cells. This process helped protect neurons from serious damage.^100^, ^101^

### 3.2 Tau pathology

Tau is an intrinsically disordered protein that contains 85 phosphorylation sites, with over 50 of these sites having been identified as being phosphorylated.^102^ The protein is predominantly expressed in neurons, where it exists in six primary isoforms.^103^ The process of phosphorylation, a posttranslational modification, is essential for the biological function of tau, which tracks axonal microtubules and enhances their stability, thereby contributing to axonal growth and transportation.^104^ However, hyperphosphorylated tau has been implicated in the formation of intracellular NFTs, a typical pathological characteristic of the brains of AD patients.^105^ Consequently, phosphorylated tau (p‐tau) is recognized as a reliable biomarker for AD diagnosis and is also considered a promising target for AD treatment.^106^

GSK3 is a kinase of significant importance for p‐tau, with approximately 30 serine or tyrosine residues from tau identified as potential phosphorylation sites for GSK3.^107^ It has also been observed that GSK3's phosphorylation of tau is correlated with the formation of tangle‐like filament morphology.^108^ Importantly, recent studies illustrate that GSK3β can directly acetylate the K15 site of tau protein, which has the negative consequence of inhibiting the ubiquitination degradation process and reducing its activity‐inhibiting state (p‐GSK3β^ser9^), ultimately causing GSK3β to become excessively activated.^109^ In essence, a detrimental cycle appears to have developed between tau pathology and GSK3 over‐activation in the brains of AD patients.

**FIGURE 3:**
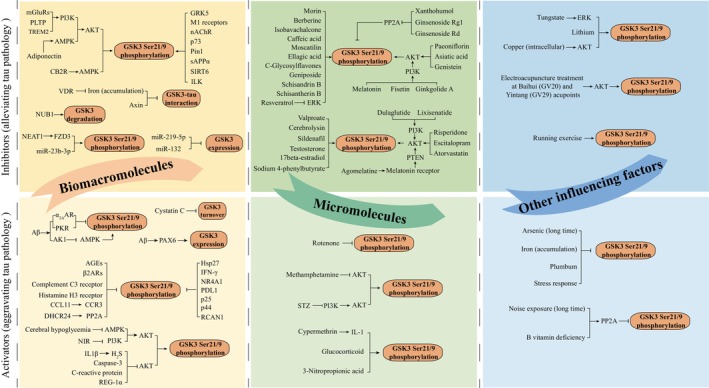
Factors influencing tau pathology through GSK3. Biomacromolecules influencing GSK3‐tau are highlighted in yellow, micromolecules in green, and other influencing factors in blue. The darker shades in the upper half signify factors that inhibit GSK3 and alleviate tau pathology, while the lighter shades in the lower half represent factors that activate GSK3 and aggravate tau pathology.

A meta‐analysis conducted on studies related to AD involving the use of 3×Tg‐AD mice found a positive correlation between Morris water maze (MWM) performance and p‐tau levels. Additionally, p‐GSK3β^Ser9^ was identified as the primary contributor.^110^ Further research involving GSK3α deficiency mice suggested that GSK3α also played a role in tau hyperphosphorylation.^111^ AChE inhibitors, such as donepezil, remain the frontline treatment for AD, scientific investigators have discovered that in AD, AChE is colocalized with p‐tau in neurofibrillary tangles. They have also found that GSK3β‐induced tau hyperphosphorylation suppressed the expression of AChE. Clinical studies involving the use of irreversible GSK3β inhibitors have reported a 35 ± 16% increase in AChE activity among patients.^112^ Here we introduce the exacerbation of tau pathology by GSK3 signaling mediated by various biomacromolecules and micromolecules (Figure 3).

#### 3.2.1 PI3K/AKT‐mediated GSK3 signaling in tau pathology

The AKT enzyme is responsible for transferring the phosphate group to Ser21/9 of GSK3, thereby inhibiting excessive activation of GSK3 and maintaining the stability of p‐tau. It has been established that AKT is initially phosphorylated and activated by phosphoinositide 3‐kinase (PI3K).^113^ Therefore, it is widely believed that the PI3K/AKT/GSK3 signaling pathway plays a crucial role in tau phosphorylation.^6^ Various upstream factors influencing PI3K or AKT may also partly affect tau phosphorylation through GSK3, which to some extent explains the interplay between neuroinflammation, neuronal apoptosis, and GSK3‐mediated tau pathology.

C‐reactive protein, an immunomodulatory protein upregulated during inflammatory responses, induces tau phosphorylation in conjunction with alterations in AKT/GSK3β.^114^ The excessive phosphorylation of tau induced by the inflammatory cytokine IL‐1β serves as a hallmark of the association between inflammation stimulation and tau pathology in AD. The phosphorylation capability of AKT on GSK3β is nullified by the sulfhydration of AKT at Cys77, which is contingent on elevated levels of intracellular hydrogen sulfide (H_2_S) provoked by IL‐1β. This phenomenon leads to tau hyperphosphorylation and synaptic dysfunction.^115^ Interestingly, a separate study demonstrated that exogenous administration of H_2_S can directly sulfhydrate GSK3β, resulting in decreased tau phosphorylation.^116^ Thus, it is evident that high levels of H_2_S have a dual impact on AKT/GSK3‐mediated tau pathology. Therefore, in an inflammatory environment, whether AKT is cysteinylated or GSK3 is cysteinylated by H_2_S, which one takes precedence, is a thought‐provoking question worthy of further exploration. The caspase family proteins, crucial proteases in cellular apoptosis, play essential roles in various biological processes in the nervous system.^117^, ^118^ Tau serves as a substrate for cysteine proteases, and caspase‐cleaved tau has been detected in the AD brain but not in the normal brain. Importantly, caspase‐cleaved tau exhibits higher fibrillogenicity compared to full‐length tau. A combination of phosphorylation events and caspase activation contributes to the process of tau oligomerization in AD.^119^ GSK3β‐mediated tau phosphorylation precedes caspase cleavage. Additionally, caspase‐3, a member of the caspase family, relieves the inhibition of GSK3β by cleaving AKT, thereby increasing the occurrence of phosphorylation events.^119^, ^120^

Impairment of insulin signaling in the brain has been associated with AD. Overexpression of Regenerating islet‐derived 1α (REG‐1α) in the brains of AD patients triggers tau phosphorylation through the AKT/GSK3 signaling pathway.^121^ Mice with neuron‐specific insulin receptor (NIR) KO exhibit activation of the PI3K/AKT/GSK3β signaling pathway and significant tau pathology.^122^ Overexpression of Phospholipid Transfer Protein (PLTP) in 3×Tg‐AD mice alleviates various AD symptoms by limiting excessive activation of GSK3β and tau hyperphosphorylation through the PI3K/AKT pathway.^123^, ^124^ Metabotropic Group I glutamate receptors (mGluRs) can influence PI3K/AKT, resulting in reduced GSK3β‐mediated tau hyperphosphorylation.^125^ The aforementioned proteins involve cellular signal transduction and intracellular and extracellular substance exchange. While a few studies have observed changes in their levels associated with alterations in the PI3K/AKT/GSK3β signaling pathway, this phenomenon is likely the result of multiple factors influencing it, such as insulin signaling, lipid transport, and metabolism. The underlying mechanisms by which these factors ultimately impact tau pathology through GSK3β remain to be elucidated.

#### 3.2.2 Aβ‐mediated GSK3 signaling in tau pathology

The investigation of the correlation between Aβ and tau has been a consistent area of focus in research on AD.^126^ GSK3 serves as a crucial element in receiving stimulation from Aβ and promoting tau pathology.^127^ The research on the interaction between Aβ and tau, as well as the role of GSK3 within it, primarily revolves around the conformation of Aβ. Aβ_1‐42_ monomers, as opposed to oligomers, induce a PHF‐like conformation of tau, resulting in multiple effects on tau, including phosphorylation, conformational changes, and degradation, while simultaneously elevating GSK3 levels.^128^ It is worth noting that Aβ_1‐42_ oligomers can also promote GSK3‐mediated tau pathology, with solubility being a crucial prerequisite.^129^ Aβ_1‐42_ oligomers can bind with tau to form soluble stable complexes, enhancing the possibility of tau receiving phosphoryl groups from GSK3β.^130^ Additionally, Aβ_1‐42_ oligomers exhibit high affinity binding with GSK3α, thereby promoting GSK3α‐catalyzed tau phosphorylation.^117^ These findings confirm the interrelationship between Aβ, tau, and GSK3, although certain details still require further investigation. For instance, if Aβ can bind to both tau and GSK3α, does that imply the formation of a complex involving all three proteins? Does the binding of Aβ to tau facilitate GSK3β‐mediated phosphorylation of tau, and does GSK3β exhibit a similar strong interaction with Aβ as GSK3α does?

In addition to directly binding to tau and promoting its phosphorylation by GSK3, the regulatory role of Aβ in GSK3 activity is equally significant. The indirect pathway of Aβ, mediated through norepinephrine α2A adrenergic receptor (α2AAR) signaling, surpasses direct GSK3β activation in terms of potency,^127^ underscoring its crucial role in the pathogenesis of AD. The cerebral neurons exhibit widespread distribution of α2AAR, playing a pivotal role in transmitting norepinephrine signaling.^118^ Aβ oligomers have the potential to bind to α2AAR and induce conformational changes, redirecting norepinephrine signaling towards GSK3β activation, thereby exacerbating tau hyperphosphorylation.^127^ Moreover, Aβ also activates GSK3 and intensifies tau pathology through the promotion of adenylate kinase 1 (AK1) and double‐stranded RNA‐dependent protein kinase (PKR).^131^, ^132^

Aβ also enhances the expression of E2F1 and c‐Myb, which activate PAX6, a transcription factor linked to brain development and overexpressed in AD patients and animal models. PAX6 promotes GSK3β transcription and elevates the p‐tau level at Ser356, Ser396, and Ser404. Besides, blocking PAX6 signaling partially mitigates Aβ‐induced neuronal death.^133^ Besides phosphorylation, Aβ also enhances tau accumulation via GSK3β/SC35 signaling, which activates tau exon 10 splicing and increases intranuclear distribution.^134^, ^135^

The role of Aβ_1‐42_ in driving GSK3‐mediated phosphorylation of p‐tau has been extensively acknowledged. The non‐amyloidogenic degradation pathway product of amyloid precursor protein (APP), known as sAPPα, exhibits a protective effect on AD pathophysiology by inhibiting GSK3β and reducing tau phosphorylation,^136^, ^137^ suggesting that other intermediate products of APP processing merit further attention in relation to their association with GSK3.

#### 3.2.3 AMPK‐mediated GSK3 signaling in tau pathology

Recent studies have indicated that AMP‐activated protein kinase (AMPK) serves as a crucial regulator of bioenergy metabolism and plays a significant role in AD.^138^ In various animal models of AD, low AMPK activity has been detected, while artificially increasing AMPK expression or activity has been found to ease tau hyperphosphorylation. Notably, GSK3β agonists wortmannin have been found to limit the positive effects of AMPK on AD, emphasizing the pivotal role of GSK3β in AMPK‐mediated suppression of p‐tau.^139^ Moreover, research has revealed that memory impairment and tau hyperphosphorylation were observed in type‐2 cannabinoid receptor (CB2R) KO mice with lower activity of AMPK alongside higher activity of GSK3β.^139^ In contrast, selective activation of CB2R was found to limit GSK3β activation and tau hyperphosphorylation; however, this protective effect was lost when AMPK was simultaneously inhibited.^140^ To summarize, the activation of AMPK inhibits GSK3β appears to be the primary mechanism by which CB2R mitigates tau pathology. Adiponectin, a hormone secreted by adipocytes, has been found to function as an euglycemic agent.^141^ Long‐term adiponectin deficiency has been associated with the induction of tau pathology and other AD pathologies.^142^ Mechanistically, adiponectin triggers AMPK phosphorylation, leading to the up‐regulation of p‐AKT and p‐GSK3β^Ser9^.^142^ Hence, adiponectin can effectively restrict tau hyperphosphorylation through the AMPK/AKT/GSK3β signaling pathway. However, it is noteworthy to mention that cerebral hypoglycemia has also been known to trigger tau pathology via the same signaling pathway, namely AMPK/AKT/GSK3β.^143^

#### 3.2.4 GSK3 signaling mediated by other proteins in tau pathology

In addition to the above few signaling pathways or stimuli, there are numerous proteins that have been reported to directly or indirectly affect GSK3‐mediated tau pathology. Firstly, there is a class of proteins that have been found to be abnormally expressed in AD patients or AD animal models, including Deficiency in G protein‐coupled receptor‐5 (GRK5), Heat shock protein 27 (Hsp27), 3‐β‐Hydroxysteroid‐Δ‐24‐reductase (DHCR24), Sirtuin 6 (SIRT6), regulator of calcineurin 1 (RCAN1), Cystatin C, and beta‐2 adrenergic receptors (β2ARs). Researchers have discovered the abnormality of GSK and p‐tau during the investigation of their roles in AD. However, there is currently a lack of evidence to directly support their involvement through the modulation of GSK3 expression or activity. Therefore, further mechanistic research is still required as substantiating evidence. Deficiency in G protein‐coupled receptor‐5 (GRK5) has been linked with mild cognitive impairment.^144^ In GRK5 KO mice, overactivation of GSK3β and tau hyperphosphorylation were observed, thus providing additional evidence of GRK5's inhibitory effect on GSK3β.^145^ Heat shock protein 27 (Hsp27) transgenic mice showed a striking pathological phosphorylation of tau, with upregulated p‐GSK3β^Tyr216^ and downregulated p‐GSK3β^Ser9^ indicating that GSK3β was the immediate cause of p‐tau.^146^ Increased expression of p44, a short isoform of p53, has been found to elevate the risk of tau pathology.^147^ Moreover, haploinsufficiency for p73, a member of the p53 family, has demonstrated a similar phenomenon.^148^ Several kinases, among them GSK3β, have been implicated in p44/p73‐related tau pathology. 3‐β‐Hydroxysteroid‐Δ‐24‐reductase (DHCR24), an enzyme that converts desmosterol to cholesterol, has been found to be down‐regulated in individuals with AD.^149^ Its levels are closely associated with caveolin‐1, an intrinsic membrane protein that is responsible for structuring caveolae leading to a key role in PP2A phosphorylation site localization. DHCR24 has been shown to promote the activity of PP2A, resulting in increased levels of p‐GSK3β^Tyr216^ and p‐tau.^150^ Sirtuin 6 (SIRT6), a “longevity gene” that optimizes energy homeostasis, is underexpressed in aging‐related diseases such as AD.^151^ SIRT6 KO mice have exhibited significant AD‐like symptoms and tau hyperphosphorylation caused by GSK3 activation.^152^ In patients with AD, the continuous overexpression of the regulator of calcineurin 1 (RCAN1) has been observed to result in the downregulation of Calcineurin, an essential phosphatase that catalyzes tau dephosphorylation. Furthermore, RCAN1 has been found to induce GSK3β‐catalyzed tau hyperphosphorylation.^153^ Cystatin C has also been observed to be upregulated in the brains of both AD patients and model animals, and its overexpression in neurons has been shown to accelerate tau phosphorylation by impeding the turnover of GSK3β.^154^ A similar increase in beta‐2 adrenergic receptors (β2ARs) has also been observed, and their stimulation has been found to influence GSK3β and Cyclin‐dependent kinase 5 (CDK5) in contributing to tau pathology.^155^, ^156^

Another major category of proteins, known as receptors, have the ability to perceive incoming signals and regulate cellular functions in response to signal stimulation. Several types of receptors have been reported, and their activation or inhibition ultimately influences GSK3‐mediated tau hyperphosphorylation. The observation was made that the activation of M1 receptors in 3×Tg‐AD mice led to a decrease in GSK3β activity and p‐tau levels. It was also noted that administration of an M1 antagonist noticeably aggravated the pathologic phosphorylation of tau.^157^ Based on these findings, M1 receptors were believed to play a critical role in the regulation of GSK3β and p‐tau. Furthermore, the Alpha7 nicotinic acetylcholine receptor (nAChR) was found to be involved in the regulation of calcium channels, which, when activated, could mitigate tau hyperphosphorylation via GSK3β,^158^ while the inhibition of the histamine H3 receptor was shown to relieve GSK3β‐related pathologic phosphorylation of tau.^159^ Complement C3 receptor has been reported to promote GSK3β activity, while its inhibition has been proven to alleviate tau hyperphosphorylation catalyzed by GSK3β.^160^ The nuclear receptor subfamily 4 group A member 1 (NR4A1) is recognized for its positive impact on synaptic plasticity and memory formation.^161^ Nonetheless, excessive expression of NR4A1 may activate GSK3β which can subsequently result in tau hyperphosphorylation.^162^ Chemokine ligand 11 (CCL11) is a well‐established inflammation marker that undergoes upregulation, and CC chemokine receptor 3 (CCR3) is the receptor for CCL11 found in hippocampal neurons.^163^, ^164^ CCR3/CCL11 has been associated with elevated p‐tau levels, and GSK3β plays a partial role in this mechanism.^164^

Other proteins that influence GSK3‐mediated tau pathology exert their effects through mechanisms that primarily involve direct binding to GSK3 or affecting the interaction between GSK3 and tau, as well as directly impacting the phosphorylation activation of GSK3, and so on. Studies have suggested that both GSK3β and tau bind to the same region of PS1, thereby promoting the enzymatic effects of GSK3β in phosphorylating its substrate, tau.^165^ Furthermore, the key tau kinase, CDK5, is generally activated by the protein p35. However, the p35 calpain cleavage product, p25, has a higher preference for binding to and activating GSK3β compared to CDK5, which leads to the phenomenon of p‐tau enhancement.^156^, ^166^ Nedd8 ultimate buster 1 (NUB1) is a protein that is induced by interferon and is known to accelerate the clearance of proteins eliminated by the ubiquitin‐proteasome system.^167^ Research indicates that NUB1 has multiple functions in regulating tau pathology, including the aggregation and phosphorylation of tau. Importantly, NUB1 promotes the degradation of GSK3β and also impedes the interaction between GSK3β and tau.^168^ Axin restricts the interaction between GSK3β and tau, which prevents GSK3β from pathologically phosphorylating tau.^169^ The programmed cell death protein 1/programmed cell death 1 ligand 1 (PD1/PDL1) has established prominence in cancer therapy.^170^ Recent studies unearthed a PDL1‐GSK3β immune complex within the brains of APP/PS1 and 5×FAD mice, an entity that heightens GSK3β activity and p‐tau levels.^171^

The preceding paragraph has indicated the potential of ILK to augment the inhibitory effects on GSK3.^7^ In N1E‐115 neuroblastoma cells, inactivation of ILK led to the loss of its inhibitory effect on GSK3β, which subsequently caused an elevation in p‐tau levels.^172^ The previous paragraph has also explained the role of GSK3 in the Wnt/β‐catenin pathway. In aged rats, inhibited Wnt signaling by Dkk‐1 decreased PP2A activity and increased GSK3β activity, which resulted in excessive tau phosphorylation at multiple sites in the hippocampus.^173^ Pin 1 is a promising target for cancer therapy.^174^ However, the inhibition of Pin1 in AD amplifies GSK3β activation and tau hyperphosphorylation.^175^ It has also been reported that GSK3β affects the proportions of different isoforms of tau mRNA through alternative splicing. Prion protein PrPC serves as an upstream regulator that inhibits GSK3β activation.^176^ Furthermore, interferon‐gamma (IFN‐γ) initiates cellular immunity and triggers tau hyperphosphorylation via GSK3β signaling.^177^

#### 3.2.5 MiRNA‐mediated GSK3 signaling in tau pathology

In brain tissues of AD patients, there is a reduced content of miR‐219‐5p, which has a negative correlation with both GSK3β and tau‐tubulin kinase 1 (TTBK1).^178^ The 3′ untranslated region of GSK3β mRNA and TTBK1 mRNA share a base sequence of 5′‐GACAAUC‐3′, which can bind with miR‐219‐5p, thereby inhibiting the expressions of GSK3β (instead of the phosphorylation of GSK3β) and TTBK1, as well as the hyperphosphorylation of tau.^178^ The most significant miRNA decrease observed in AD patients' neurons is miR‐132,^179^, ^180^ whose down‐regulation is closely linked to the pathological processes of AD, such as the formation of NFTs.^181^ Mechanistically, miR‐132 can regulate the expression and various post‐translational modifications, including phosphorylation and acetylation, and limit the phosphorylation of tau through its negative role in both the transcription and translation of GSK3β.^179^ The phenomenon of decreased miR‐23b‐3p has been observed in the plasma of AD patients, cortex of APP/PS1 mice, brain of SAMP8 mice, and SH‐SY5Y/APPswe cells. Delivery of miR‐23b‐3p into the ventricles of APP/PS1 mouse brains can improve their cognitive deficits, alleviate AD pathology, and especially reduce tau phosphorylation at multiple sites. Mechanistic studies indicate that miR‐23b‐3p mainly exerts its effects by reducing the levels and activity of GSK3β.^182^ Moreover, the LncRNA nuclear paraspeckles assembly transcript 1 (NEAT1) facilitates the transcription of frizzled class receptor 3 (FCR3), thus limiting the phosphorylation of tau by GSK3β.^183^

#### 3.2.6 Micromolecules and other stimulus mediated GSK3 signaling in tau pathology

Inducers of disease models, environmental pollutants, and certain physical factors like noise pollution are all significant contributors to tau pathology that cannot be overlooked. The link between AD and diabetes is widely recognized, with GSK3 playing a crucial role.^7^, ^184^ Glucocorticoids, the key hormone responsible for raising blood glucose levels, have also been reported to activate GSK3β, elevate p‐tau levels, and impair memory in db/db mice.^185^ As an inducer of sporadic AD models, STZ is frequently used in the modeling of both AD and diabetes mellitus.^186^ Experiments have shown that STZ can stimulate tau hyperphosphorylation through the PI3K/AKT/GSK3 signaling pathway.^186^, ^187^ Rotenone, a renowned compound extracted from Derris known for its ability to induce apoptosis, is also frequently employed in the establishment of PD models, has been demonstrated to elevate p‐tau levels through the activation of GSK3β.^188^

Methamphetamine abuse often leads to dementia, and experimental evidence has shown that methamphetamine contributes to tau hyperphosphorylation through the AKT/GSK3β signaling pathway.^189^ Cypermethrin, a pesticide, can up‐regulate GSK3β. Initially, cypermethrin blocks the heparin‐binding epidermal growth factor (HB‐EGF) signaling pathway, which results in neuroinflammation. The excessive generation of IL‐1 ultimately increases the levels of GSK3β and p‐tau.^190^ Furthermore, 3‐Nitropropionic acid triggers the mitochondrial origin of oxidative stress, while excessive activation of GSK3β and tau hyperphosphorylation were also observed in the hippocampus and cortex of mice exposed to 3‐nitropropionic acid.^191^

Prolonged exposure to arsenic can elevate GSK3β activity and exacerbate tau hyperphosphorylation in SH‐SY5Y cells, while multiple GSK3β inhibitors can alleviate the effects of arsenic.^192^ Plumbum exposure can also lead to tau hyperphosphorylation, with the activation of GSK3β and CDK5 being the declared pathogenesis.^193^ Furthermore, iron accumulation can increase the activities of GSK3β and CDK5, thereby worsening tau pathology.^194^ Paricalcitol can alleviate iron accumulation by activating the vitamin D receptor (VDR), thus down‐regulating p‐GSK3β^Tyr216^ and ultimately preventing hyperphosphorylation of tau at Ser396 and Thr181 sites.^195^ Intranasal administration of deferoxamine produces similar effects on GSK3β and tau.^194^ Chronic noise exposure has also been linked to tau pathology, with PP2A/GSK3β being under‐regulated.^196^ Interestingly, B‐vitamin deficiency has been found to exhibit a similar function as noise.^197^ Moreover, stress responses such as electric shocks and ether anesthesia can exacerbate the pathologic phosphorylation of tau via GSK3β.^198^, ^199^

These studies suggest the crucial significance of maintaining a healthy lifestyle in preventing the occurrence and progression of AD symptoms. It is also advisable for AD patients to eliminate or minimize negative neurostimulants such as environmental pollutants and drugs while undergoing treatment. Nevertheless, further research is still required to substantiate the direct association between these stimulating factors and GSK3‐mediated tau pathology. This is because, based on existing research, their impact on the nervous system is intricate and potentially irreversible, with GSK3‐mediated tau pathology likely representing just one of the most overt pathological outcomes.

### 3.3 Amyloid pathology

Just like GSK3 is associated with other typical pathological features, amyloid pathology is also known to be linked with it. This pathology occurs due to an accumulation of Aβ, which is cleaved from its precursor protein APP. APP is an integral membrane protein that is abundantly expressed in the synapses of neurons and plays vital roles in synapse formation and repair, anterograde neuronal transport, and iron export.^200^ There are three isoforms of APP that mainly concentrate in different tissues or cells. These isoforms are named APP_695_, APP_751_, and APP_770_, based on the number of amino acid residues. The shortest isoform, APP_695_, lacks a Kunitz‐type protease inhibitor sequence in the ectodomain and is the most abundant in brains and neurons.^200^, ^201^ However, APP_751_ and APP_770_ are expressed more abundantly in platelets and peripheral cells.^200^

**FIGURE 4:**
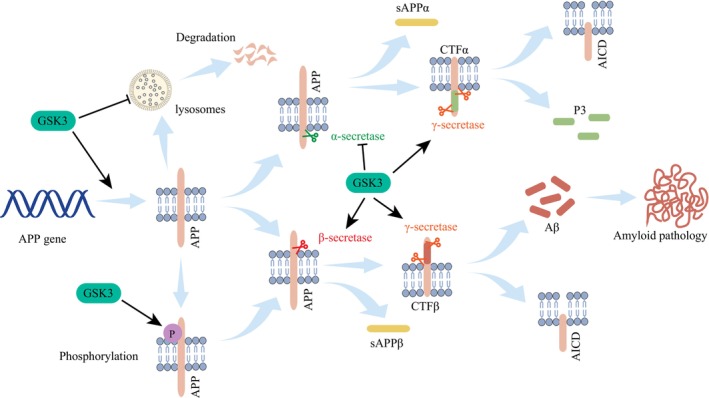
The impact of GSK3 on the process of APP cleavage. GSK3 modulates the cleavage of APP by regulating α‐secretase, β‐secretase, and γ‐secretase activities. It also exerts influence over the expression, phosphorylation, and degradation of APP.

It should be noted that not all cleavage processes of APP lead to the production of Aβ. Depending on whether or not Aβ is produced, cleavage processes can be classified into non‐amyloidogenic pathways and amyloidogenic pathways. Figure 4 shows that in the non‐amyloidogenic pathway, APP is firstly cleaved by α‐secretase to form sAPPα and α‐C terminal fragment (CTFα).^200^ Unlike Aβ, sAPPα plays crucial roles in neuroprotection, synaptic plasticity related learning and memory,^136^ and it is produced more along with increased neuronal activity.^202^ CTFα can be further cleaved to P3 and the APP intracellular domain (AICD), catalyzed by γ‐secretase.^200^ The amyloidogenic pathway, on the other hand, involves β‐secretase instead of α‐secretase. In this pathway, APP is initially cleaved by α‐secretase to form soluble amyloid precursor protein beta (sAPPβ) and β‐C terminal fragment (CTFβ). The γ‐secretase also catalyzes CTFβ cleavage, leading to the generation of AICD. However, under further processing by γ‐secretase at multiple sites, 40 amino acid Aβ_1‐40_ and the 42 amino‐acid Aβ_1‐42_ are ultimately generated.^200^ These highly neurotoxic peptide fragments are the inducers of amyloid pathology. Therefore, it is significant to reduce Aβ generation by suppressing β‐secretase and γ‐secretase activities. Additionally, activating α‐secretase could be helpful.

GSK3β is involved in various stages of Aβ formation, both directly and indirectly. These stages include its impact on APP expression, phosphorylation, lysosomal degradation, as well as its influence on α‐secretase, β‐secretase, and γ‐secretase (Figure 4). The association between GSK3 activation and Aβ pathology has been observed in numerous cellular or animal models. CDK5 knockdown in 3×Tg‐AD mice executed at the age of 15 months, showed significant reduction in amyloid plaques. The activation of GSK3β and PP2A was declared to be the primary reason.^203^ The author of this report primarily focuses on the association between CDK5 and GSK3β. Unfortunately, the direct cause of GSK3β activation leading to the formation of amyloid plaques in this one of the most classic AD animal models has not been thoroughly investigated. Nevertheless, GSK3β siRNA was employed to knockdown GSK3β in STZ‐induced, in vitro and in vivo models of AD. The remarkable outcomes were overcome down‐regulation of Aβ in cell models and mitigation of Aβ accumulation in the cortex and hippocampus of animal models. Inhibition of APP and β‐secretase 1 (BACE1) by GSK3β siRNA was found to be the crucial factor contributing to this phenomenon.^204^ Conversely, inhibition of β‐secretase was observed to stimulate GSK3β and intensify tau hyperphosphorylation.^205^ Senescence‐accelerated prone mouse strain 8 (SAMP8) mouse model is a form of rapid aging animal models associated with cognitive impairment. In SAMP8 mice, the modification of the PI3K/AKT pathway gradually activated GSK3β with age. Ultimately, both β‐secretase and γ‐secretase were activated, leading to an increase in the levels of Aβ_1‐40_ and Aβ_1‐42_ in platelet and hippocampi.^206^

Hence, the impact on β‐secretase and γ‐secretase appears to be crucial in linking GSK3 with Aβ pathology, and several literature reports provide strong evidence supporting this inference. As a kinase of glycogen synthase, the over‐activation of GSK3 is one of the causes of glucose metabolism disorder leading to diabetes. Advanced glycation end products (AGEs) are toxic substances generated in the body after long‐term hyperglycemia.^7^ Receptor for AGEs (RAGE) had been established to contribute to amyloid pathology. GSK3β and p38 were identified as the connecting links between RAGE signal and Aβ. Gene silencing of RAGE curtailed GSK3β and ameliorated Aβ accumulation, accompanied by attenuated activities of β‐secretase and γ‐secretase.^207^ Through the association of T‐cell factor‐4 with the BACE1 promoter, the Wnt/β‐catenin pathway could impede BACE1 transcription. It must be noted that the GSK3 inhibitor propelled Wnt signaling and concurrently repressed BACE1 transcription.^208^

The transcriptional expression, phosphorylation, and degradation processes of APP also exert an influence on the ultimate accumulation of Aβ, wherein GSK plays a significant role. When comparing gene expression between normal people, patients with mild cognitive impairment, and AD patients, GSK3β expression was positively linked to the expression of Amyloid beta precursor protein binding family B member 2,^209^ a transcription activator of APP.^210^ In an inflammatory milieu, astrocytes have the potential to transmit CK1 to neurons via extracellular vesicles, which finally exacerbates the formation of amyloid plaques, where GSK3 has a definitive function. More specifically, in response to the stimulation of IL‐1β, astrocytes secrete extracellular vesicles that envelop CK1. Then, after the transfer of CK1 to neurons, it engages in a complex with APC and GSK3, which obstructs the degradation of β‐catenin. Consequently, this escalates the level of APP mRNA and in turn, intensifies the co‐localization of BACE1 and APP. In essence, GSK3 is instrumental in regulating APP expression and amyloid processing through this pathway.^211^ Suppressing GSK3β also diminished APP phosphorylation,^212^ which might change the APP cleavage process and stop Aβ production.^213^ Besides, restraining GSK3 fostered the nuclear translocation of the transcription factor EB, thereby enhancing the number of lysosomes, which ultimately improved APP autophagic degradation, consequently curtailing Aβ level.^214^ Apart from curbing Aβ accumulation, GSK3β also influenced the sensitivity of the hippocampus towards Aβ. It was found that the voluntary running of mice prevented their hippocampal network from disturbance caused by Aβ, and this defense was dependent on the inhibition of GSK3β.^215^

### 3.4 Oxidative stress and neuroinflammation

Efforts have been made in recent decades to alleviate amyloid pathology and tau pathology in AD, but drug development progress has been slow.^216^ Therefore, researchers have also shown great interest in other pathogenesis, such as oxidative stress and neuroinflammation in AD. Elevated reactive oxygen species (ROS), mitochondrial dysfunction, lipid peroxidation, and ferroptosis are common phenomena observed in AD patients or animal models, thus characterizing oxidative stress.^21^ Inflammation, as indicated by the high levels of inflammatory cytokines TNF‐α, IL‐6, and others found in AD patients' serum and brain, has also been linked to AD. The alteration of immune cells, such as reactive astrocytes, T cells, and particularly activated microglia in the brain of AD patients, explains the occurrence of inflammation at the cellular level.^217^

**FIGURE 5:**
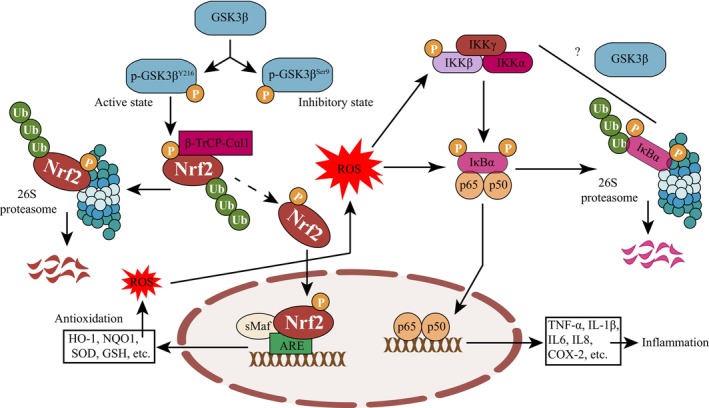
Effects of GSK3β on cellular oxidative stress and inflammation via Nrf2 and NF‐κB. Activated GSK3β phosphorylates Nrf2, ultimately leading to its ubiquitination and degradation. Consequently, the reduced levels of Nrf2 weaken the transcriptional expression of cellular antioxidant enzymes, resulting in excessive accumulation of ROS. Excessive ROS triggers the degradation of IκBα, leading to the release of NF‐κB (p65 and p50) into the nucleus, thereby upregulating the transcriptional expression of pro‐inflammatory factors and exacerbating inflammation. However, the direct impact of GSK3β on IKK and IκBα remains uncertain.

The role of GSK3 in oxidative stress and neuroinflammation has been implicated in its known substrate nuclear factor erythroid 2‐related factor 2 (Nrf2), a significant transcription factor that responds to environmental stimuli, particularly elevated ROS.^218^ Nrf2 transfers from the cytoplasm into the nucleus to perform its biological functions. The dimerization of the transcriptional co‐activator sMaf stabilizes Nrf2 in the nucleus, allowing it to interact with Antioxidant Response Element (ARE) to enhance transcription of various genes (Figure 5), such as heme oxygenase‐1 (HO‐1), NAD(P)H:quinone oxidoreductase 1 (NQO1), superoxide dismutase (SOD), etc.^219^

As a sophisticated regulator, the content of Nrf2, particularly the content in the nucleus, is tightly controlled by the ubiquitination system. Kelch‐like ECH‐associated protein 1 (Keap1) functions to concatenate Nrf2 and Cul3‐Rbx1, a ubiquitin ligase complex, ultimately leading to ubiquitination and degradation of Nrf2.^220^ However, Keap1 is not the only ubiquitination process involved in Nrf2 degradation. As shown in the Figure 5, another ubiquitin ligase complex, β‐TrCP‐Cul1, is also responsible for Nrf2 ubiquitination and degradation. Crucially, when p‐GSK3β^Y216^ phosphorylates Nrf2, it greatly promotes the negative regulatory process of Nrf2.^218^ Therefore, suppressing GSK3β is considered a promising therapeutic strategy for AD due to its Nrf2 activation effect. For example, an antisense oligonucleotide of GSK3β that reduced the level of GSK3β in the cortex of SAMP8 mice also exhibited Nrf2 activation and improved learning and memory abilities.^221^ GSK3 has also been found to be involved in the muscarinic M1 receptor‐induced Nrf2 accumulation in the nucleus. Overexpression of the M1 receptor in PC12 cells enhanced the level of Nrf2, activated the antioxidant response element (ARE), and facilitated the transcription and translation of HO‐1. GSK3β(Δ9) nullified the Nrf2‐regulated oxidation while GSK3β(Y216F) had little to no effect on it.^222^

The activity of Nrf2 is closely linked with inflammation, largely due to its crosstalk with NF‐κB, a well‐known regulator of inflammation.^223^ As shown in Figure 5, excessive production of ROS would facilitate multiple steps of NF‐κB activation, which also relies on upstream regulatory factor IKKα ubiquitination and degradation.^223^ Although there is currently no evidence of GSK3 involvement in IKKα ubiquitination, the collective suppression of GSK3β and NF‐κB has been observed in some cells and animal models.^224^, ^225^ For instance, soluble epoxide hydrolase (sEH) is a promising target for inflammatory and AD treatment.^226^ Inhibiting sEH in Aβ_1‐42_ induced AD mice was found to stabilize epoxyeicosatrienoic acids, a series of compounds with anti‐inflammatory properties. Interestingly, a high level of p‐GSK3β^Ser9^, accompanied by Nrf2 activation and NF‐κB inhibition, was also observed.^227^

From the perspective of signaling pathways, the Nrf2 and NF‐κB signaling pathways are crucial for the neuroinflammation caused by excessive activation of GSK3. Meanwhile, from the perspective of neural cells, both microglia and astrocyte activation have been found to be associated with GSK3. Microglia, which are resident immune cells in the brain, release a large amount of inflammatory factors after M1 polarization in pathological conditions. Similarly, activated astrocytes can also release inflammatory factors and chemokines to attract microglia into the damaged area. Aβ_1‐42_ induces neuronal apoptosis and releases phosphatidylserine, thereby activating microglia and promoting neuroinflammation. The GSK3/Wnt signaling pathway plays a crucial role in this process, inhibiting PI3K/AKT to activate GSK3β and facilitate microglial activation.^228^ A low level of DHCR24 was accompanied by GSK3β activation and hyperphosphorylation of tau.^149^, ^150^ In addition to tau pathology, overexpression of DHCR24 in BV‐2 cells reversed the microglia polarization trend induced by Aβ_25‐35_. Increased levels of arginase‐1, IL‐4, and TGF‐β, and decreased levels of iNOS, IL‐1β, and TNF‐α reflected that DHCR24 promoted M2 polarization of microglia, playing an anti‐inflammatory role. The key mechanism is the activation of AKT and thereby the inhibition of GSK3β.^229^ GSK3β is also involved in microglial activation via phosphorylation of astrocyte CCAAT enhancer‐binding protein delta (CEBPD), a well‐known participant in inflammation‐related diseases.^230^ In astrocytes of AD patients, excessive activation of the calcium‐activated potassium channel KCa3.1 was observed. In LPS‐induced neuroinflammation mice, the KO of KCa3.1 protected neurons and alleviated cognitive impairment. KCa3.1 deficiency mice showed activation of the PI3K/AKT pathway, suppression of GSK3β, and inhibition of NF‐κB signaling.^231^ Moreover, in vitro cultures of various glial cells extracted from rat cortex released various inflammatory factors including IL‐1β, TNF‐α, and IL‐10, which were significantly decreased by different GSK3 inhibitors.^232^

### 3.5 Mitochondrial dysfunction

Mitochondria serve as the primary sites for cellular energy metabolism. The brain, being the most metabolically active organ, relies on the abundant ATP produced through mitochondrial oxidative phosphorylation. In the brain of AD patients, noticeable alterations occur in both the structure and function of mitochondria, including changes in glucose and oxygen metabolism due to respiratory chain dysfunction, as well as accumulation of ROS and mutations in mitochondrial DNA.^233^

Under oxidative stress conditions, GSK3β translocates from the cytoplasm to the mitochondria in a kinase activity and voltage‐dependent anion channel 2 (VDAC2)‐dependent manner. This process is associated with increased cell death and can be blocked by GSK3β inhibitors.^234^ Studies have demonstrated that GSK3β inhibition can enhance mitochondrial respiration and membrane potential, while altering NAD(P)H metabolism. These metabolic effects are associated with the increased stability of PGC‐1α protein, enhanced nuclear localization, and increased co‐transcriptional activation. This suggests that the GSK3β/PGC‐1α axis may play an important role in maintaining neuronal metabolic integrity.^235^ Dynamin‐related protein 1 (Drp1) is a key protein involved in mitochondrial division and is highly expressed in neuronal cells. Research has shown that GSK3β can induce the phosphorylation of Drp1, resulting in mitochondrial fragmentation and increased neuronal sensitivity to Aβ toxicity. In vivo and in vitro models have shown that limiting the phosphorylation of Drp1 induced by GSK3β is an effective way to protect the nervous system from Aβ damage.^236^ Axonal transport defects caused by Aβ result in impaired mitochondrial transport, leading to acute impairments in mitochondrial trafficking in Aβ‐stimulated hippocampal neurons. Researchers have found that the activation of GSK3β by Aβ is a significant contributor to mitochondrial transport dysfunction.^237^ Another study has revealed that GSK3β can co‐localize with and regulate HDAC6 phosphorylation in hippocampal neurons. HDAC6 regulates tubulin acetylation and affects the function of the motor protein‐1, playing a crucial role in the anterograde transport of mitochondria within axons. Serotonin, a neuromodulator, can enhance mitochondrial transport through AKT‐mediated inhibition of GSK3β, indicating that GSK3β may interfere with mitochondrial transport by affecting HDAC6 activity.^238^

### 3.6 Autophagy impairment

Autophagy is a cellular catabolic process responsible for breaking down damaged organelles and protein aggregates through the lysosomal pathway. It helps to clear out Aβ and p‐tau.^22^ The mTOR, which is regulated by GSK3, plays a crucial role in promoting autophagy, thereby mitigating the pathological effects of Aβ and p‐tau.^239^ In the previous chapter, the relationship between the low expression of DHCR24 and the pathogenesis of AD, specifically tau hyperphosphorylation and microglia M1 polarization was presented. DHCR24 knockdown resulted in overactivation of GSK3β, which is a significant molecular mechanism. Additionally, DHCR24 knockdown SH‐SY5Y cells showed autophagy inhibition characterized by decreased autophagosome. Mechanistically, the activated GSK3β phosphorylates mTOR at Ser2448, stimulating it to act as an autophagy inhibitor.^240^ PS1 KO human neural stem cells also displayed GSK3β related autophagy suppression.^241^

## 4 THERAPEUTIC STRATEGIES FOR AD TARGETING GSK3

**FIGURE 6:**
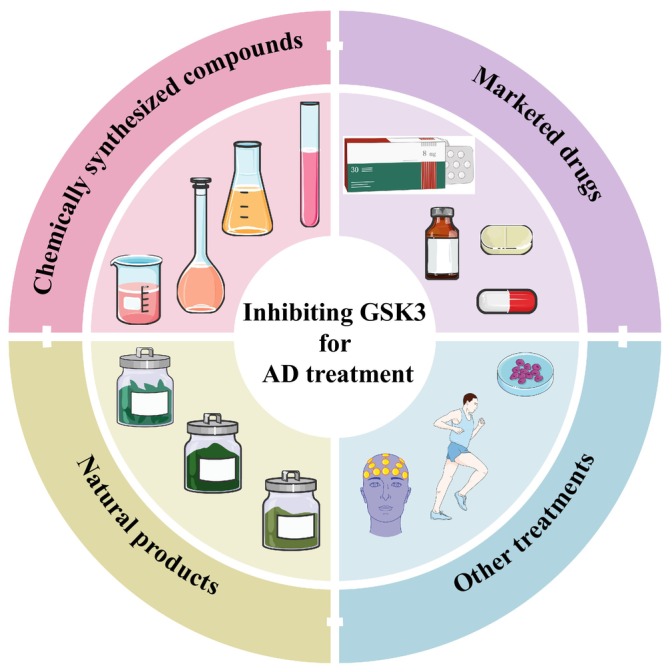
Pharmacotherapy and additional interventions for AD employing GSK3 inhibition as a strategic approach.

As previously stated, the excessive activation of GSK3 in AD patients is closely related to tau phosphorylation, Aβ pathology, neuroinflammation, oxidative stress damage, and other factors contributing to AD. Therefore, targeting GSK3 has been a major focus of AD treatment strategies. In this section, we will systematically summarize preclinical experiments that have exhibited GSK3 inhibition and AD therapeutic effects, including newly developed GSK3‐targeting compounds, natural products discovered from traditional herbal medicine, and clinically approved drugs (Figure 6). Meanwhile, we also present an overview of other therapeutic approaches that can impinge upon the activity of GSK3 (Figure 6).

### 4.1 Chemically synthesized targeted therapeutics

Using chemical synthesis to obtain compounds targeting the desired protein such as GSK3 has always been a direct and effective method.^242^, ^243^ This article summarizes more than 10 new GSK3‐targeting compounds, which have shown certain efficacy in AD model animals. Their chemical structures and biological functions are summarized in Table 1. Compound 47 is an imidazo[1,2‐b]pyridazine that exhibits remarkable GSK3β and p‐tau inhibition activities, with an IC_50_ of 0.37 nM and 58 nM for GSK3β and p‐tau inhibition, respectively.^245^ As a designed GSK3β inhibitor, compound 10a has shown comprehensive anti‐AD effects in AlCl_3_ combined with d‐galactose induced AD mice, including improved cognitive ability, reduced tau hyperphosphorylation, and lowered hippocampal neuronal damage, among others.^246^ In addition to the aforementioned compound designs that target GSK3β alone, many pharmaceutical chemists have shifted their focus towards the design of AD drugs targeting multiple targets, including GSK3β. Compound 11c is a dual inhibitor designed based on GSK3β and AchE, which has shown objective recovery of learning and memory ability in scopolamine‐ or Aβ_1‐42_‐induced AD mice. The neuroprotective effects of Compound 11c include inhibiting brain tau phosphorylation and increasing acetylcholine and serotonin levels in the brain.^247^ Another GSK3β, AchE dual inhibitor Compound GT15 can reduce tau phosphorylation induced by okadaic acid and ROS induced by LPS in vitro. In in vivo experiments, GT15 can also enhance the learning and memory ability of AD mice.^250^ Compound LDC8 is a dual inhibitor of GSK3β and CDK5, which can alleviate neuronal inflammation damage. In the zebrafish model induced by Aβ_1‐42_, LDC8 therapy increased synaptic density by 67.6%.^249^ Compound 2 is a synthetic BACE1/GSK3β dual inhibitor, which exhibits good inhibitory effects on Aβ formation, alleviating inflammation damage, and promoting neurogenesis.^251^

**TABLE 1:** Information on chemically synthesized GSK3 inhibitors.

Compounds	Models	Biological activities	Ref
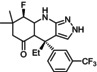 Compound 1	Rats at post‐natal day 10	Alleviate tau hyperphosphorylation	244
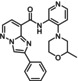 Compound 47	3×Tg‐AD mice	Alleviate tau hyperphosphorylation	245
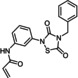 Compound 10a	AlCl_3_ combined with d‐galactose induced AD mice	Improve learning and memory Alleviate tau hyperphosphorylation Reduce hippocampal neurons damage	246
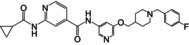 Compound 11c	Scopolamine induced ICR mice Aβ_1‐42_ induced ICR mice Aβ_1‐42_ induced SD rats	Improve learning and memory Inhibit AChE activity in brain	247
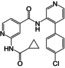 Compound 33	3×Tg‐AD mice	Alleviate tau hyperphosphorylation	248
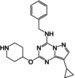 Compound LDC8	Aβ_1‐42_ induced zebrafish	Reduce synaptic degeneration	249
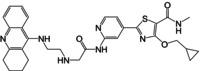 Compound GT15	Scopolamine induced ICR mice	Improve learning and memory Alleviate tau hyperphosphorylation	250
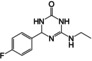 Compound 2	H4‐APPswe cell line LPS induced primary rat microglia and astrocyte cells	Reduce inflammation of nerve cells	251
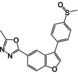 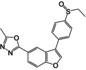 Compound (S)‐9b and (S)‐9c	Cold water stress mice	Alleviate tau hyperphosphorylation	252
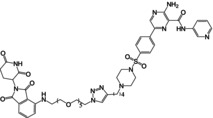 Compound PT‐65	Aβ_1‐42_ induced SH‐SY5Y cells okadaic acid induced SD rats	Relieve cell damage Alleviate tau hyperphosphorylation Improve learning and memory	253
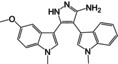 Compound 6 h	LPS induced C57/B6J mice	Reduce microglial activation and astrocyte proliferation	254

Regrettably, there is currently limited research on PROTAC targeting GSK3, as well as scarce reports on chemical drug designs focusing on GSKα. PROTAC, which enables targeted compound ubiquitination degradation, represents a novel and promising strategy in drug design. And compounds targeting GSKα provide an alternative option for inhibiting GSK3 in the treatment of AD. Compound PT‐65 is a PROTAC, which has strong affinity for both GSK3α and GSK3β. By degradation of GSK3, PT‐65 can inhibit tau hyperphosphorylation and protect against cognitive impairments.^253^ Compound 1 is a recently reported rare inhibitor specifically designed for GSK3α. This compound and its derivatives designed based on it exhibit a selectivity for GSK3α that is significantly higher than that for GSK3β, up to 37 times higher. In addition, Compound 1 has also demonstrated good activity in inhibiting tau phosphorylation both in vitro and in vivo.^244^

### 4.2 Natural products

Searching for drugs or lead compounds from natural products has always been an important avenue for drug development. Many researchers have also attempted to identify natural products with therapeutic potential for AD and elucidate their mechanisms, based on the long‐term experience of using traditional Chinese medicine or herbal remedies. The involvement of GSK3 in various pathological aspects of AD is evident. It is apparent that these natural compounds exert their effects on GSK3, thereby protecting neurons, alleviating tau pathology, reducing neuroinflammation, and mitigating oxidative stress damage.

By stimulating PI3K/AKT, selenomethionine effectively suppressed GSK3β and triggered Wnt signaling in the hippocampus. This resulted in a significant increase in the number of differentiated neurons from neural stem cells, coupled with an obvious decrease in astrocytes.^255^ In separate studies, 20(S)‐protopanaxadiol and oleanolic acid improved the learning and memory abilities of APP/PS1 mice by inhibiting GSK3β and activating Wnt/β‐catenin in the hippocampus, ultimately enhancing neurogenesis.^256^ Similarly, icarisid II exerted a similar molecular mechanism and promoted the proliferation of neural stem cells and neuronal differentiation, leading to considerable neurogenic benefits.^257^, ^258^

Morin is a natural flavonoid that impacts tau hyperphosphorylation by inhibiting GSK3β, as suggested by both the crystal structure of complex (PDB: 1H8F) and surface plasmon resonance result.^259^ Moreover, it has been observed that morin binds to the ATP binding pocket of GSK3β with a 67 μM dissociation constant value.^260^ Ginsenoside Rg1 has shown significant efficacy in various animal models of dementia, such as OKA‐induced AD rats, aluminum chloride‐induced AD mice and Squirrel monkeys.^261^, ^262^, ^263^ Although the detailed mechanisms revealed in different reports are not completely the same, Ginsenoside Rg1 is believed to inhibit GSK3 activity in all cases. For instance, in the AD mouse model, the inhibition of GSK3 by Ginsenoside Rg1 is accompanied by a decrease in the level of PP2A. In the Squirrel monkey model, the author suggests that the Wnt/GSK‐3β/β‐catenin pathway is the main signal pathway under regulation, and that neurocyte apoptosis, oxidative stress damage and inflammatory reactions are all inhibited.^261^, ^262^, ^263^ Along similar molecular mechanism, ginsenoside Rd significantly attenuated tau pathology.^264^ Lignans obtained from Schisandra chinensis Turcz. (Baill.) have a novel dibenzocyclooctadiene skeletal structure and considerable biological activities.^223^ Among them, schisandrin B and schisantherin B exhibited substantial potential in alleviating tau pathology, and their roles in inhibiting GSK3β were also identified.^265^, ^266^ Furthermore, several other natural products including paeoniflorin,^267^ genistein,^231^ berberine,^268^ isobavachalcone,^269^ moscatilin,^270^ xanthohumol,^271^ resveratrol,^272^ fisetin,^273^ C‐glycosylflavones,^274^ geniposide,^275^, ^276^, ^277^ ginkgolide A,^278^ caffeic acid,^279^ asiatic acid,^280^ ellagic acid,^281^ etc., also played crucial roles in inhibiting GSK3 and mitigating tau hyperphosphorylation, and the specific molecular mechanisms were illustrated in Figure 3.

Certain natural compounds have demonstrated favorable anti‐inflammatory and antioxidant activities in both in vivo and in vitro models of AD. Oxyphylla A effectively suppressed high ROS levels in N2a/APP cells, enhanced the learning and memory abilities of SAMP8 mice, and mitigated oxidative stress damage. Its effect on the GSK3β/Nrf2 pathway was identified as the key underlying molecular mechanism, whereby oxyphylla A triggered AKT phosphorylation, leading to the inactivation of GSK3β and ultimately activating Nrf2.^282^ Similarly, thymol and artemether, two other natural products also activated Nrf2, albeit through different pathways. Thymol achieved GSK3β/Nrf2 activation via AKT, while artemether acted via AMPK.^283^, ^284^ Eupatin is a natural substance that has the ability to bind with GSK3β and hinder its activity. In mouse microglia cells and macrophages that were treated with lipopolysaccharides (LPS), eupatin was observed to diminish both the overall content as well as the level of p65 phosphorylation.^224^ Anthocyanins were also investigated for their effects on LPS‐induced neuroinflammation mice. It was discovered that, besides regulating AKT, GSK3β, and NF‐κB, anthocyanins were able to decrease ROS in the hippocampus of these mice.^285^ Additionally, another study reported that in APP/PS1 mice, anthocyanins exhibited antioxidant abilities through the GSK3β/Nrf2 pathway.^286^ Neuroprotectin D1 is a pro‐resolving mediator produced from docosahexaenoic acid in neuronal cells. In N2a/APPswe cells, neuroprotectin D1 inhibited GSK3β and led to enhanced autophagy with decreased expression of beclin 1.^287^ Amentoflavone has been found to inhibit Aβ_1‐42_‐induced neuronal apoptosis both in vitro and in vivo. In vivo studies have shown that Amentoflavone can increase neuronal activity in the hippocampal region of AD rats and alleviate cognitive impairment at an epigenetic level. In vitro experiments using SH‐SY5Y cells with AMPKα knockdown demonstrated the importance of AMPK in the biological functions of Amentoflavone. Mechanistically, amentoflavone acting via the AMPK pathway inhibits GSK3β and exerts anti‐apoptotic and anti‐AD effects.^288^ Meridianins are indole alkaloids isolated from marine ascidians, which display remarkable cognitive improvement in 5×FAD mice by inhibiting GSK3β and rescuing synaptic loss as well as suppressing neuroinflammation.^289^

As mentioned in paragraph 4 of Section 3.3, GSK3β is also involved in glucose metabolism, and AGEs are formed after chronic hyperglycemia.^7^, ^290^ AGEs increased GSK3β activity and tau phosphorylation in female APP/PS1 mice. Treatment with trehalose mitigated the negative effect of AGEs through down‐regulation of GSK3β and triggered nuclear translocation of transcription factor EB (TFEB).^291^ Similarly, Calycosin exhibits a neuroprotective effect against AGEs‐induced neurotoxicity by inhibiting GSK3β. In a rat model of diabetes complicated with AD, Calycosin improves learning and memory, and reduces AD‐related pathologies such as neurofibrillary tangles and amyloid deposits, as well as mitochondrial dysfunction.^292^

Finally, other natural products such as galangin,^293^ naringenin,^294^ wogonin,^295^ curcumin,^296^ and linarin^297^ have exhibited certain GSK3 inhibitory activity and neuroprotective effects related to AD in vitro. However, these effects have not yet been validated by in vivo experiments.

### 4.3 Marketed drugs

The secondary development of clinical drugs is an important aspect that cannot be ignored, with aspirin being the classic success story in this regard. Hence, many drugs already on the market have been investigated for their potential to relieve AD pathology related to GSK3β. The antidepressant fluoxetine inhibited GSK3β activity through PP2A, thereby activating the Wnt/β‐catenin pathway. As a result, 3×Tg‐AD mice treated with fluoxetine exhibited reduced neuronal apoptosis, synaptic damage, and Aβ accumulation.^298^ Sodium selenate also activated the Wnt/β‐catenin pathway via PP2A and GSK3β and effectively alleviated the symptoms of 3×Tg‐AD mice.^299^ Rapamycin is a well‐known mTOR inhibitor and autophagy inducer with proven efficacy in clearing toxic proteins such as Aβ and p‐tau. Researchers have found that Rapamycin can increase Wnt3a expression, resulting in GSK3β inhibition and increased β‐catenin, thereby improving AD pathology.^300^

Lithium is a recognized inhibitor of GSK3 and is one of the most widely used mood stabilizers for the treatment of bipolar affective disorder. It has also been attempted to treat various GSK3‐related diseases.^2^, ^301^ The relationship between lithium and GSK3, as well as its role in AD, have also been reported. For instance, lithium can dose‐dependently reduce the mRNA levels of GSK3 in the cerebral cortex and hippocampal neurons of rats, and its inhibition of GSK3β can promote AchE degradation by proteasomes and activate cholineacetyltransferase. In animal models of AD, Lithium has also been demonstrated to alleviate scopolamine‐induced memory impairment.^302^, ^303^ LiCl also improved the level of Nrf2 in APP/PS1 mice, upregulated the levels of antioxidant enzymes such as SOD and GSH‐Px, downregulated the level of lipid peroxidation product MDA in both brains and serum, further indicating the antioxidative effects of LiCl via the GSK3β/Nrf2 pathway.^304^ A retrospective cohort study identified a correlation between the usage of lithium and a reduced risk of developing dementia. This clinical evidence validates the role of lithium and further supports the potential of GSK3 as a therapeutic target for AD.^305^

Melatonin, an amine hormone produced by the pineal gland, has gained recognition for its hypnotic effects, although it is currently only approved as a drug in some countries. Melatonin can inhibit GSK3β through the PI3K/AKT signaling pathway and exert a series of anti‐AD effects such as inhibiting p‐tau.^306^ The GSK3β inhibition effect of melatonin can also lead to the downregulation of ADAM10 through the NF‐κB signal and the upregulation of BACE1 and PS1.^307^ In N2a cells induced by okadaic acid, the addition of melatonin can decrease GSK3β mRNA levels, increase nuclear localization of Nrf2, and reduce the expression of many anti‐inflammatory factors.^308^ Overall, melatonin may play a significant role in inhibiting both the expression and phosphorylation activation of GSK3β.

Moreover, certain drugs have exhibited promising results in alleviating GSK3‐induced tau hyperphosphorylation. Figure 3 compiles these drugs, primarily medications for neurological disorders such as agomelatine,^309^ risperidone,^310^ escitalopram,^311^, ^312^ valproate,^313^ and cerebrolysin.^314^ It also includes other classes of drugs such as dulaglutide,^315^ lixisenatide,^316^ atorvastatin,^317^ sildenafil,^318^ 17beta‐estradiol,^319^ allantoin,^320^ and sodium 4‐phenylbutyrate.^321^

### 4.4 Other treatments

Mesenchymal stem cell transplantation is a clinically objective and low‐side‐effect approach for treating diseases. In attempts to treat AD with mesenchymal stem cell transplantation, researchers have discovered numerous effects of the approach, including promoting lysosomal‐autophagic clearance of Aβ and p‐tau aggregates, inducing microglial M2 polarization, reducing MDA levels, enhancing neuronal dendrite growth, and reducing synaptic losses, among others. Mechanistically, mesenchymal stem cells regulate miR‐134 levels by activating SIRT1, thereby inhibiting GSK3β.^322^ In clinical‐grade human umbilical cord mesenchymal stem cells (hUC‐MSCs), significant restoration of cognitive function was observed in SAMP8 mice. In an AD cellular model, hUC‐MSCs secreted hepatocyte growth factor (HGF) that targeted the cMet‐AKT‐GSK3β signaling pathway, downregulated hyperphosphorylated tau protein, reversed spine loss, and increased synaptic plasticity.^323^ In another study using human fetal neuroepithelial cells (hNSCs) obtained from the endbrain of human fetuses aborted at 13 weeks and implanted in AD mice, TrkA/B phosphorylation in AD mice was significantly increased, which led to increased phosphorylation levels of AKT and GSK3β(Ser9). The inhibition of GSK3β was accompanied by decreases in p‐tau, BACE1, Aβ, deactivation of microglia, and reduced inflammatory cytokine secretion.^324^

Exogenous administration of BDNF or neurotrophic peptidergic compounds is another emerging therapeutic approach for AD. Injection of BDNF into the hippocampus can enhance learning and memory in Okadaic acid‐induced AD rats. Mechanistically, BDNF is thought to act on the PI3K/AKT pathway, inhibiting GSK3β activity and down‐regulating the phosphorylation of tau protein at multiple sites, including p‐tau (Thr231), and p‐tau (Ser396/404).^325^ A neurotrophic peptidergic compound, known as P021, has been reported to promote BDNF expression and exhibit a significant inhibitory effect on GSK3β in 3×Tg‐AD mice. At the epigenetic level, P021 improves cognitive impairment, reduces tau hyperphosphorylation and amyloid plaque deposition, and promotes neurogenesis and synaptic plasticity in AD mice.^326^ (D‐Ser2)Oxm is a peptide hormone and growth factor similar to Oxyntomodulin that activates glucagon‐like peptide 1 and glucagon receptors. In APP/PS1 mice, the administration of (D‐Ser2)Oxm significantly increased p‐PI3K and p‐AKT expression in the hippocampus while reducing p‐GSK3β‐Y216 levels. Based on this potential mechanism, (D‐Ser2)Oxm not only alleviated working memory and long‐term spatial memory impairments, but also reduced the number of Aβ plaques in the hippocampus and reversed inhibition of LTP at the hippocampal synapses.^327^

Electroacupuncture has a long history in traditional Chinese medicine and recent years have witnessed an increasing focus on its role in regulating nerve signal transduction and related diseases.^328^ In experiments involving APP/PS1 mice, electroacupuncture treatment at Baihui (GV20) and Yintang (GV29) acupoints has been shown to improve cognition while observably increasing p‐AKT^Ser437^, p‐GSK3β^Ser9^, and p‐tau, emphasizing the importance of electroacupuncture on AKT/GSK3β/tau signaling.^329^ Additionally, high‐frequency (50 Hz) electroacupuncture at GV20 and BL23 was found significantly enhancing learning and memory ability in AD rats with Aβ_1‐42_ induction. This treatment also inhibited GSK3β, leading to increased synaptic curvature, reduced synaptic cleft width, increased postsynaptic density, and apparent neuroprotective effects against neuronal damage.^330^ Moreover, for d‐galactose‐induced AD rats, preventive electroacupuncture at GV20‐BL23 acupoints resulted in GSK3β inhibition, down‐regulation of p‐mTOR, and ultimately enhanced autophagy.^331^ Regular physical exercise is beneficial for human health, and running is considered an effective way to combat the AD process. In APP/PS1 mice, treadmill exercise for 5 months has been shown to inhibit GSK3 and p‐tau but have no effect on CDK5.^332^

## 5 DISCUSSION AND PERSPECTIVES

GSK3 is a serine/threonine kinase that comprises two isoforms, GSK3α and GSK3β, and serves as a fundamental regulatory factor in many vital life processes owing to its ability to phosphorylate numerous substrate proteins. Clinical evidence, AD animal models, and some AD related cell models all demonstrate varying degrees of GSK3 abnormalities. On one hand, the expression levels of GSK3 in the brains of AD patients and AD model animals are significantly higher than those in normal individuals and animals. On the other hand, these highly expressed GSK3 also exhibit relatively higher levels of activation, most notably reflected in the reduced proportion of inhibitory states (p‐GSK3αSer21 and p‐GSK3βSer9).

The abundant expression of GSK3 and the reduced proportion of its inhibitory states play various significant negative roles in AD. Firstly, neuronal damage (Figure 2) includes neuronal proliferation decrease, neuronal maturation retardation and loss of immature neurons, as well as abnormalities in synaptic, axonal, dendritic spine structures, and functions. Importantly, as a kinase, GSK3 can catalyze phosphorylation at approximately 30 sites in tau. The relationship between GSK3 and p‐tau is most widely reported, with various AD‐related pathologies such as Aβ or signaling pathways such as PI3K/AKT signaling and AMPK signaling ultimately leading to GSK3‐mediated tau hyperphosphorylation (Figure 3). Additionally, GSK3 is involved in various processes of APP formation of Aβ (Figure 4), promoting the expression of the APP, inhibiting lysosomal degradation of APP, leading to an increased potential for the formation of Aβ precursor protein. Phosphorylation of APP, inhibition of α‐secretase, activation of β‐secretase and γ‐secretase directly advance the process from APP to Aβ. GSK3 is also involved in oxidative stress and neuroinflammation in AD mainly through Nrf2 signaling and NF‐κB signaling (Figure 5). GSK3 primarily increases the ubiquitination and degradation of Nrf2 through phosphorylation it, while its effects on NF‐κB are reported to be indirect, such as through the impact of increased ROS. Other AD‐related pathological processes such as mitochondrial dysfunction and autophagy impairment also involve the participation of GSK3.

There have been many studies on GSK3 inhibitors, but most of them are preclinical studies. Even the well‐known GSK3 inhibitor lithium lacks sufficient clinical evidence to prove its effectiveness in treating AD. Therefore, many synthetic compounds not only target GSK3 as a single target, such as GSK3β/AchE inhibitors and BACE1/GSK3β inhibitors, have shown certain anti‐AD effects in vivo and in vitro. Many natural products can inhibit GSK3 activity, but most lack sufficient experimental evidence to prove their direct inhibitory effects. Morin is the only one reported to bind to GSK3β and inhibit its activity, thus showing anti‐AD effects. In terms of marketed drugs, besides lithium, melatonin, escitalopram, and others have been reported to have some impact on GSK3 activity in AD. In addition to drug treatments, alternative therapies such as electroacupuncture and emerging therapeutic approaches like mesenchymal stem cell transplantation may also have positive effects on AD, possibly related to GSK3.

Considering the lack of clinically proven effective GSK3‐targeted drugs for AD, we present some perspectives and recommendations herein with the hope of facilitating future research related to GSK3 and AD.

1. Differences in functionality between GSK3α and GSK3β. The cellular localization and brain distribution of GSK3α and GSK3β differ, hence their roles in AD may also vary. Drug development research has primarily concentrated on the functions and inhibition of GSK3β, as the crystal structure of GSK3α remains unclear. Nevertheless, recently designed compounds targeting GSK3α have shown significant therapeutic effects on AD. The aforementioned evidence suggests that researchers now need to give equal attention to GSK3α as they do to GSK3β.
2. The relationship between conformational changes in GSK3 and its substrate selectivity. The conformational transitions of GSK3 play a decisive role in the execution of its functions. However, current research has only focused on the activation or inhibition of GSK3 activity. Clarifying the relationship between conformational changes in GSK3 and its substrate selectivity can aid in the more targeted design of therapeutic drugs targeting GSK3 and help avoid unnecessary side effects.
3. The role of GSK3 in the crosstalk among various pathologies in AD. It is widely acknowledged that complex crosstalk exists between different pathologies in AD. While GSK3 has been shown to exacerbate multiple AD pathologies, its role in the crosstalk among these pathologies remains partially understood. For instance, while it is known that Aβ activates GSK3β indirectly by activating α2AAR, the feedback regulatory effect of p‐tau on GSK3 remains unclear. The role of GSK3 in the crosstalk between oxidative damage and neuroinflammation has been partially elucidated (Figure 5). However, it is yet to be explored whether GSK3 has a direct activating effect on NF‐κB or if it exerts an indirect influence solely through the regulation of ROS.
4. The role of GSK3 in other neuronal cells apart from neurons. Existing research has found that GSK3 is overexpressed or hyperactivated in the entire brain region of AD patients or animal models. However, current studies primarily focus on GSK3 in neurons. As research progresses, increasing evidence suggests that other types of neural cells, such as microglia, astrocytes, and oligodendrocytes, also play a crucial role in the occurrence and development of AD. Therefore, it remains to be explored whether there are significant changes in the expression levels or activity of GSK3 in these neural cells during AD, as well as the impact of GSK3 on their respective unique functions.
5. Novel GSK3 inhibitors designed using updated methodologies. Present chemically synthesized GSK3 inhibitors mainly focus on inhibiting target proteins by combining them. PROTAC technology that prioritizes ubiquitination and degradation of target proteins is becoming more widely used as drug chemistry advances. Currently, only one study has been reported on GSK3β‐treated AD using PROTACs. However, we believe this is an excellent attempt, and we anticipate that novel structured GSK3 inhibitors will exhibit potential in anti‐AD medication.
6. Combination therapy of GSK3 inhibitors with other drugs. Considering that existing GSK3 inhibitors are still in the preclinical stage, there may be limitations in developing therapeutic drugs solely targeting GSK3. Furthermore, certain compounds targeting both GSK3 and other proteins such as AchE, CDK5, and BACE1 have shown potential for AD treatment. These findings provide some insights into combination therapy for AD, exploring whether the strategy of using GSK3 inhibitors in conjunction with existing AD drugs like donepezil and lecanemab can better control the progression of AD. This represents a meaningful endeavor.

## CONFLICT OF INTEREST STATEMENT

The authors declare that there are no conflicts of interest.

## Data Availability

Data sharing is not applicable to this review article as no new data were created or analyzed in this review manuscript.

## References

[1] Embi N , Rylatt DB , Cohen P . Glycogen synthase kinase‐3 from rabbit skeletal muscle. Separation from cyclic‐AMP‐dependent protein kinase and phosphorylase kinase. Eur J Biochem. 1980;107(2):519‐527. 6249596

[2] Beurel E , Grieco SF , Jope RS . Glycogen synthase kinase‐3 (GSK3): regulation, actions, and diseases. Pharmacol Therapeut. 2015;148:114‐131. 10.1016/j.pharmthera.2014.11.016 PMC4340754 25435019

[3] Cormier KW , Woodgett JR . Recent advances in understanding the cellular roles of GSK‐3. F1000Res. 2017;6:F1000. Faculty Rev‐167. 10.12688/f1000research.10557.1 PMC5321126 28299185

[4] Hermida MA , Dinesh Kumar J , Leslie NR . GSK3 and its interactions with the PI3K/AKT/mTOR signalling network. Adv Biol Regul. 2017;65:5‐15. 28712664 10.1016/j.jbior.2017.06.003

[5] Woodgett JR . Molecular cloning and expression of glycogen synthase kinase‐3/factor A. EMBO J. 1990;9(8):2431‐2438. 2164470 10.1002/j.1460-2075.1990.tb07419.x PMC552268

[6] Kaidanovich‐Beilin O , Woodgett JR . GSK‐3: functional insights from cell biology and animal models. Front Mol Neurosci. 2011;4:40. 22110425 10.3389/fnmol.2011.00040 PMC3217193

[7] Wang L , Li J , Di LJ . Glycogen synthesis and beyond, a comprehensive review of GSK3 as a key regulator of metabolic pathways and a therapeutic target for treating metabolic diseases. Med Res Rev. 2022;42(2):946‐982. 34729791 10.1002/med.21867 PMC9298385

[8] Ali A , Hoeflich KP , Woodgett JR . Glycogen synthase kinase‐3: properties, functions, and regulation. Chem Rev. 2001;101(8):2527‐2540. 11749387 10.1021/cr000110o

[9] Azoulay‐Alfaguter I , Yaffe Y , Licht‐Murava A , et al. Distinct molecular regulation of glycogen synthase kinase‐3alpha isozyme controlled by its N‐terminal region: functional role in calcium/calpain signaling. J Biol Chem. 2011;286(15):13470‐13480. 21266584 10.1074/jbc.M110.127969 PMC3075693

[10] Rippin I , Eldar‐Finkelman H . Mechanisms and therapeutic implications of GSK‐3 in treating neurodegeneration. Cells. 2021;10(2):262. 33572709 10.3390/cells10020262 PMC7911291

[11] Diehl JA , Cheng M , Roussel MF , Sherr CJ . Glycogen synthase kinase‐3beta regulates cyclin D1 proteolysis and subcellular localization. Genes Dev. 1998;12(22):3499‐3511. 9832503 10.1101/gad.12.22.3499 PMC317244

[12] Bijur GN , Jope RS . Proapoptotic stimuli induce nuclear accumulation of glycogen synthase kinase‐3 beta. J Biol Chem. 2001;276(40):37436‐37442. 11495916 10.1074/jbc.M105725200 PMC1973163

[13] Yao Q , Long C , Yi P , et al. C/EBPbeta: a transcription factor associated with the irreversible progression of Alzheimer's disease. CNS Neurosci Ther. 2024;30(4):e14721. 38644578 10.1111/cns.14721 PMC11033503

[14] Shanks HRC , Chen K , Reiman EM , et al. p75 neurotrophin receptor modulation in mild to moderate Alzheimer disease: a randomized, placebo‐controlled phase 2a trial. Nat Med. 2024;30(5). 10.1038/s41591-024-02977-w PMC11186782 38760589

[15] 2022 Alzheimer's disease facts and figures. Alzheimers Dement. 2022;18(4):700‐789. 35289055 10.1002/alz.12638

[16] Tan CC , Yu JT , Wang HF , et al. Efficacy and safety of donepezil, galantamine, rivastigmine, and memantine for the treatment of Alzheimer's disease: a systematic review and meta‐analysis. J Alzheimers Dis. 2014;41(2):615‐631. 24662102 10.3233/JAD-132690

[17] Howard R , McShane R , Lindesay J , et al. Donepezil and memantine for moderate‐to‐severe Alzheimer's disease. N Engl J Med. 2012;366(10):893‐903. 22397651 10.1056/NEJMoa1106668

[18] Sevigny J , Chiao P , Bussiere T , et al. The antibody aducanumab reduces Abeta plaques in Alzheimer's disease. Nature. 2016;537(7618):50‐56. 27582220 10.1038/nature19323

[19] Knopman DS , Jones DT , Greicius MD . Failure to demonstrate efficacy of aducanumab: an analysis of the EMERGE and ENGAGE trials as reported by Biogen, December 2019. Alzheimers Dement. 2021;17(4):696‐701. 33135381 10.1002/alz.12213

[20] Irwin MR , Vitiello MV . Implications of sleep disturbance and inflammation for Alzheimer's disease dementia. Lancet Neurol. 2019;18(3):296‐306. 30661858 10.1016/S1474-4422(18)30450-2

[21] Misrani A , Tabassum S , Yang L . Mitochondrial dysfunction and oxidative stress in Alzheimer's disease. Front Aging Neurosci. 2021;13:617588. 33679375 10.3389/fnagi.2021.617588 PMC7930231

[22] Zhang W , Xu CC , Sun JC , Shen HM , Wang JG , Yang CB . Impairment of the autophagy‐lysosomal pathway in Alzheimer's diseases: pathogenic mechanisms and therapeutic potential. Acta Pharm Sin B. 2022;12(3):1019‐1040. 35530153 10.1016/j.apsb.2022.01.008 PMC9069408

[23] Liang J , Wang Y , Liu B , et al. Deciphering the intricate linkage between the gut microbiota and Alzheimer's disease: elucidating mechanistic pathways promising therapeutic strategies. CNS Neurosci Ther. 2024;30(4):e14704. 38584341 10.1111/cns.14704 PMC10999574

[24] Jakaria M , Belaidi AA , Bush AI , Ayton S . Ferroptosis as a mechanism of neurodegeneration in Alzheimer's disease. J Neurochem. 2021;159(5):804‐825. 34553778 10.1111/jnc.15519

[25] Yao HB , Shaw PC , Wong CC , Wan DC . Expression of glycogen synthase kinase‐3 isoforms in mouse tissues and their transcription in the brain. J Chem Neuroanat. 2002;23(4):291‐297. 12048112 10.1016/s0891-0618(02)00014-5

[26] Perez‐Costas E , Gandy JC , Melendez‐Ferro M , Roberts RC , Bijur GN . Light and electron microscopy study of glycogen synthase kinase‐3beta in the mouse brain. PLoS One. 2010;5(1):e8911. 20111716 10.1371/journal.pone.0008911 PMC2811740

[27] Hanger DP , Hughes K , Woodgett JR , Brion JP , Anderton BH . Glycogen synthase kinase‐3 induces Alzheimer's disease‐like phosphorylation of tau: generation of paired helical filament epitopes and neuronal localisation of the kinase. Neurosci Lett. 1992;147(1):58‐62. 1336152 10.1016/0304-3940(92)90774-2

[28] Sayas CL , Avila J . GSK‐3 and tau: a key duet in Alzheimer's disease. Cells. 2021;10(4):721. 33804962 10.3390/cells10040721 PMC8063930

[29] Pal D , Mukherjee S , Song IH , Nimse SB . GSK‐3 inhibitors: a new class of drugs for Alzheimer's disease treatment. Curr Drug Targets. 2021;22(15):1725‐1737. 33459229 10.2174/1389450122666210114095307

[30] Takahashi M , Tomizawa K , Kato R , et al. Localization and developmental changes of tau protein kinase I/glycogen synthase kinase‐3 beta in rat brain. J Neurochem. 1994;63(1):245‐255. 7515946 10.1046/j.1471-4159.1994.63010245.x

[31] Kim WY , Zhou FQ , Zhou J , et al. Essential roles for GSK‐3s and GSK‐3‐primed substrates in neurotrophin‐induced and hippocampal axon growth. Neuron. 2006;52(6):981‐996. 17178402 10.1016/j.neuron.2006.10.031 PMC4167845

[32] Jung EM , Ka M , Kim WY . Loss of GSK‐3 causes abnormal astrogenesis and behavior in mice. Mol Neurobiol. 2016;53(6):3954‐3966. 26179612 10.1007/s12035-015-9326-8 PMC4716001

[33] Kim WY , Wang X , Wu Y , et al. GSK‐3 is a master regulator of neural progenitor homeostasis. Nat Neurosci. 2009;12(11):1390‐1397. 19801986 10.1038/nn.2408 PMC5328673

[34] Morgan‐Smith M , Wu Y , Zhu X , Pringle J , Snider WD . GSK‐3 signaling in developing cortical neurons is essential for radial migration and dendritic orientation. eLife. 2014;3:e02663. 25073924 10.7554/eLife.02663 PMC4109311

[35] MacAulay K , Doble BW , Patel S , et al. Glycogen synthase kinase 3alpha‐specific regulation of murine hepatic glycogen metabolism. Cell Metab. 2007;6(4):329‐337. 17908561 10.1016/j.cmet.2007.08.013

[36] Zhou J , Freeman TA , Ahmad F , et al. GSK‐3alpha is a central regulator of age‐related pathologies in mice. J Clin Invest. 2013;123(4):1821‐1832. 23549082 10.1172/JCI64398 PMC3613907

[37] Bhattacharjee R , Goswami S , Dey S , et al. Isoform‐specific requirement for GSK3alpha in sperm for male fertility. Biol Reprod. 2018;99(2):384‐394. 29385396 10.1093/biolre/ioy020 PMC6692850

[38] Kaidanovich‐Beilin O , Lipina TV , Takao K , et al. Abnormalities in brain structure and behavior in GSK‐3alpha mutant mice. Mol Brain. 2009;2:35. 19925672 10.1186/1756-6606-2-35 PMC2785804

[39] Mukai F , Ishiguro K , Sano Y , Fujita SC . Alternative splicing isoform of tau protein kinase I/glycogen synthase kinase 3beta. J Neurochem. 2002;81(5):1073‐1083. 12065620 10.1046/j.1471-4159.2002.00918.x

[40] Soutar MP , Kim WY , Williamson R , et al. Evidence that glycogen synthase kinase‐3 isoforms have distinct substrate preference in the brain. J Neurochem. 2010;115(4):974‐983. 20831597 10.1111/j.1471-4159.2010.06988.x

[41] Wood‐Kaczmar A , Kraus M , Ishiguro K , Philpott KL , Gordon‐Weeks PR . An alternatively spliced form of glycogen synthase kinase‐3beta is targeted to growing neurites and growth cones. Mol Cell Neurosci. 2009;42(3):184‐194. 19607922 10.1016/j.mcn.2009.07.002

[42] Fuster‐Matanzo A , Llorens‐Martin M , Sirerol‐Piquer MS , Garcia‐Verdugo JM , Avila J , Hernandez F . Dual effects of increased glycogen synthase kinase‐3beta activity on adult neurogenesis. Hum Mol Genet. 2013;22(7):1300‐1315. 23257288 10.1093/hmg/dds533

[43] Morales‐Garcia JA , Luna‐Medina R , Alonso‐Gil S , et al. Glycogen synthase kinase 3 inhibition promotes adult hippocampal neurogenesis in vitro and in vivo. ACS Chem Nerosci. 2012;3(11):963‐971. 10.1021/cn300110c PMC3503340 23173075

[44] Garrido JJ , Simon D , Varea O , Wandosell F . GSK3 alpha and GSK3 beta are necessary for axon formation. FEBS Lett. 2007;581(8):1579‐1586. 17391670 10.1016/j.febslet.2007.03.018

[45] Lucas FR , Salinas PC . WNT‐7a induces axonal remodeling and increases synapsin I levels in cerebellar neurons. Dev Biol. 1997;192(1):31‐44. 9405095 10.1006/dbio.1997.8734

[46] Jiang H , Guo W , Liang X , Rao Y . Both the establishment and the maintenance of neuronal polarity require active mechanisms: critical roles of GSK‐3beta and its upstream regulators. Cell. 2005;120(1):123‐135. 15652487 10.1016/j.cell.2004.12.033

[47] Kerkela R , Kockeritz L , Macaulay K , et al. Deletion of GSK‐3beta in mice leads to hypertrophic cardiomyopathy secondary to cardiomyoblast hyperproliferation. J Clin Invest. 2008;118(11):3609‐3618. 18830417 10.1172/JCI36245 PMC2556242

[48] Hoeflich KP , Luo J , Rubie EA , Tsao MS , Jin O , Woodgett JR . Requirement for glycogen synthase kinase‐3beta in cell survival and NF‐kappaB activation. Nature. 2000;406(6791):86‐90. 10894547 10.1038/35017574

[49] O'Brien WT , Harper AD , Jove F , et al. Glycogen synthase kinase‐3beta haploinsufficiency mimics the behavioral and molecular effects of lithium. J Neurosci. 2004;24(30):6791‐6798. 15282284 10.1523/JNEUROSCI.4753-03.2004 PMC5328671

[50] Beaulieu JM , Zhang X , Rodriguiz RM , et al. Role of GSK3 beta in behavioral abnormalities induced by serotonin deficiency. Proc Natl Acad Sci U S A. 2008;105(4):1333‐1338. 18212115 10.1073/pnas.0711496105 PMC2234138

[51] Kimura T , Yamashita S , Nakao S , et al. GSK‐3beta is required for memory reconsolidation in adult brain. PLoS One. 2008;3(10):e3540. 18958152 10.1371/journal.pone.0003540 PMC2568810

[52] Beaulieu JM , Sotnikova TD , Yao WD , et al. Lithium antagonizes dopamine‐dependent behaviors mediated by an AKT/glycogen synthase kinase 3 signaling cascade. Proc Natl Acad Sci U S A. 2004;101(14):5099‐5104. 15044694 10.1073/pnas.0307921101 PMC387380

[53] Omata N , Chiu CT , Moya PR , et al. Lentivirally mediated GSK‐3beta silencing in the hippocampal dentate gyrus induces antidepressant‐like effects in stressed mice. Int J Neuropsychopharmacol. 2011;14(5):711‐717. 20604988 10.1017/S1461145710000726 PMC3125712

[54] Pandey GN , Dwivedi Y , Rizavi HS , et al. GSK‐3beta gene expression in human postmortem brain: regional distribution, effects of age and suicide. Neurochem Res. 2009;34(2):274‐285. 18584322 10.1007/s11064-008-9770-1

[55] Shahab L , Plattner F , Irvine EE , Cummings DM , Edwards FA . Dynamic range of GSK3alpha not GSK3beta is essential for bidirectional synaptic plasticity at hippocampal CA3‐CA1 synapses. Hippocampus. 2014;24(12):1413‐1416. 25208523 10.1002/hipo.22362 PMC4258099

[56] Singh SA , Winter D , Bilimoria PM , Bonni A , Steen H , Steen JA . FLEXIQinase, a mass spectrometry‐based assay, to unveil multikinase mechanisms. Nat Methods. 2012;9(5):504‐508. 22484849 10.1038/nmeth.1970 PMC3595540

[57] Dajani R , Fraser E , Roe SM , et al. Crystal structure of glycogen synthase kinase 3 beta: structural basis for phosphate‐primed substrate specificity and autoinhibition. Cell. 2001;105(6):721‐732. 11440715 10.1016/s0092-8674(01)00374-9

[58] ter Haar E , Coll JT , Austen DA , Hsiao HM , Swenson L , Jain J . Structure of GSK3beta reveals a primed phosphorylation mechanism. Nat Struct Biol. 2001;8(7):593‐596. 11427888 10.1038/89624

[59] Dajani R , Fraser E , Roe SM , et al. Structural basis for recruitment of glycogen synthase kinase 3beta to the axin‐APC scaffold complex. EMBO J. 2003;22(3):494‐501. 12554650 10.1093/emboj/cdg068 PMC140752

[60] Stamos JL , Chu ML , Enos MD , Shah N , Weis WI . Structural basis of GSK‐3 inhibition by N‐terminal phosphorylation and by the Wnt receptor LRP6. eLife. 2014;3:e01998. 24642411 10.7554/eLife.01998 PMC3953950

[61] Frame S , Cohen P , Biondi RM . A common phosphate binding site explains the unique substrate specificity of GSK3 and its inactivation by phosphorylation. Mol Cell. 2001;7(6):1321‐1327. 11430833 10.1016/s1097-2765(01)00253-2

[62] Hughes K , Nikolakaki E , Plyte SE , Totty NF , Woodgett JR . Modulation of the glycogen synthase kinase‐3 family by tyrosine phosphorylation. EMBO J. 1993;12(2):803‐808. 8382613 10.1002/j.1460-2075.1993.tb05715.x PMC413270

[63] Sutherland C , Cohen P . The alpha‐isoform of glycogen synthase kinase‐3 from rabbit skeletal muscle is inactivated by p70 S6 kinase or MAP kinase‐activated protein kinase‐1 in vitro. FEBS Lett. 1994;338(1):37‐42. 8307153 10.1016/0014-5793(94)80112-6

[64] Domoto T , Pyko IV , Furuta T , et al. Glycogen synthase kinase‐3beta is a pivotal mediator of cancer invasion and resistance to therapy. Cancer Sci. 2016;107(10):1363‐1372. 27486911 10.1111/cas.13028 PMC5084660

[65] Ding Q , Xia W , Liu JC , et al. Erk associates with and primes GSK‐3beta for its inactivation resulting in upregulation of beta‐catenin. Mol Cell. 2005;19(2):159‐170. 16039586 10.1016/j.molcel.2005.06.009

[66] Thornton TM , Pedraza‐Alva G , Deng B , et al. Phosphorylation by p38 MAPK as an alternative pathway for GSK3beta inactivation. Science. 2008;320(5876):667‐670. 18451303 10.1126/science.1156037 PMC2597039

[67] Lochhead PA , Kinstrie R , Sibbet G , Rawjee T , Morrice N , Cleghon V . A chaperone‐dependent GSK3beta transitional intermediate mediates activation‐loop autophosphorylation. Mol Cell. 2006;24(4):627‐633. 17188038 10.1016/j.molcel.2006.10.009

[68] Hernandez F , Langa E , Cuadros R , Avila J , Villanueva N . Regulation of GSK3 isoforms by phosphatases PP1 and PP2A. Mol Cell Biochem. 2010;344(1–2):211‐215. 20652371 10.1007/s11010-010-0544-0

[69] Dewachter I , Ris L , Jaworski T , et al. GSK3beta, a centre‐staged kinase in neuropsychiatric disorders, modulates long term memory by inhibitory phosphorylation at serine‐9. Neurobiol Dis. 2009;35(2):193‐200. 19379814 10.1016/j.nbd.2009.04.003

[70] Kettunen P , Larsson S , Holmgren S , et al. Genetic variants of GSK3B are associated with biomarkers for Alzheimer's disease and cognitive function. J Alzheimers Dis. 2015;44(4):1313‐1322. 25420549 10.3233/JAD-142025

[71] Platenik J , Fisar Z , Buchal R , et al. GSK3beta, CREB, and BDNF in peripheral blood of patients with Alzheimer's disease and depression. Prog Neuropsychopharmacol Biol Psychiatry. 2014;50:83‐93. 24334212 10.1016/j.pnpbp.2013.12.001

[72] Lucas JJ , Hernandez F , Gomez‐Ramos P , Moran MA , Hen R , Avila J . Decreased nuclear beta‐catenin, tau hyperphosphorylation and neurodegeneration in GSK‐3beta conditional transgenic mice. EMBO J. 2001;20(1–2):27‐39. 11226152 10.1093/emboj/20.1.27 PMC140191

[73] Lee SJ , Chung YH , Joo KM , et al. Age‐related changes in glycogen synthase kinase 3beta (GSK3beta) immunoreactivity in the central nervous system of rats. Neurosci Lett. 2006;409(2):134‐139. 17046157 10.1016/j.neulet.2006.09.026

[74] Wen Y , Planel E , Herman M , et al. Interplay between cyclin‐dependent kinase 5 and glycogen synthase kinase 3 beta mediated by neuregulin signaling leads to differential effects on tau phosphorylation and amyloid precursor protein processing. J Neurosci. 2008;28(10):2624‐2632. 18322105 10.1523/JNEUROSCI.5245-07.2008 PMC6671193

[75] Rodriguez‐Matellan A , Avila J , Hernandez F . Overexpression of GSK‐3beta in adult Tet‐OFF GSK‐3beta transgenic mice, and not during embryonic or postnatal development, induces tau phosphorylation, neurodegeneration and learning deficits. Front Mol Neurosci. 2020;13:561470. 33013321 10.3389/fnmol.2020.561470 PMC7511757

[76] Sirerol‐Piquer M , Gomez‐Ramos P , Hernandez F , et al. GSK3beta overexpression induces neuronal death and a depletion of the neurogenic niches in the dentate gyrus. Hippocampus. 2011;21(8):910‐922. 20575007 10.1002/hipo.20805

[77] Albeely AM , Williams OOF , Perreault ML . GSK‐3beta disrupts neuronal oscillatory function to inhibit learning and memory in male rats. Cell Mol Neurobiol. 2022;42(5):1341‐1353. 33392916 10.1007/s10571-020-01020-z PMC11421759

[78] Xu XF , Wang YC , Zong L , Chen ZY , Li Y . Elevating integrin‐linked kinase expression has rescued hippocampal neurogenesis and memory deficits in an AD animal model. Brain Res. 2018;1695:65‐77. 29787769 10.1016/j.brainres.2018.05.024

[79] Cuesto G , Jordan‐Alvarez S , Enriquez‐Barreto L , Ferrus A , Morales M , Acebes A . GSK3beta inhibition promotes synaptogenesis in drosophila and mammalian neurons. PLoS One. 2015;10(3):e0118475. 25764078 10.1371/journal.pone.0118475 PMC4357437

[80] Zhu LQ , Wang SH , Liu D , et al. Activation of glycogen synthase kinase‐3 inhibits long‐term potentiation with synapse‐associated impairments. J Neurosci. 2007;27(45):12211‐12220. 17989287 10.1523/JNEUROSCI.3321-07.2007 PMC6673262

[81] Hooper C , Markevich V , Plattner F , et al. Glycogen synthase kinase‐3 inhibition is integral to long‐term potentiation. Eur J Neurosci. 2007;25(1):81‐86. 17241269 10.1111/j.1460-9568.2006.05245.x

[82] Kailainathan S , Piers TM , Yi JH , et al. Activation of a synapse weakening pathway by human Val66 but not Met66 pro‐brain‐derived neurotrophic factor (proBDNF). Pharmacol Res. 2016;104:97‐107. 26687096 10.1016/j.phrs.2015.12.008 PMC4773404

[83] Jo J , Whitcomb DJ , Olsen KM , et al. Abeta(1‐42) inhibition of LTP is mediated by a signaling pathway involving caspase‐3, Akt1 and GSK‐3beta. Nat Neurosci. 2011;14(5):545‐547. 21441921 10.1038/nn.2785

[84] Gupta S , Yadav K , Mantri SS , Singhal NK , Ganesh S , Sandhir R . Evidence for compromised insulin signaling and neuronal vulnerability in experimental model of sporadic Alzheimer's disease. Mol Neurobiol. 2018;55(12):8916‐8935. 29611103 10.1007/s12035-018-0985-0

[85] DaRocha‐Souto B , Coma M , Perez‐Nievas BG , et al. Activation of glycogen synthase kinase‐3 beta mediates beta‐amyloid induced neuritic damage in Alzheimer's disease. Neurobiol Dis. 2012;45(1):425‐437. 21945540 10.1016/j.nbd.2011.09.002 PMC3694284

[86] Gan KJ , Akram A , Blasius TL , et al. GSK3beta impairs KIF1A transport in a cellular model of Alzheimer's disease but does not regulate motor motility at S402. eNeuro. 2020;7(6):ENEURO.0176‐20.2020. 10.1523/ENEURO.0176-20.2020 PMC7768277 33067366

[87] Vossel KA , Xu JC , Fomenko V , et al. Tau reduction prevents Abeta‐induced axonal transport deficits by blocking activation of GSK3beta. J Cell Biol. 2015;209(3):419‐433. 25963821 10.1083/jcb.201407065 PMC4427789

[88] Kanaan NM , Morfini GA , LaPointe NE , et al. Pathogenic forms of tau inhibit kinesin‐dependent axonal transport through a mechanism involving activation of axonal phosphotransferases. J Neurosci. 2011;31(27):9858‐9868. 21734277 10.1523/JNEUROSCI.0560-11.2011 PMC3391724

[89] Cuchillo‐Ibanez I , Seereeram A , Byers HL , et al. Phosphorylation of tau regulates its axonal transport by controlling its binding to kinesin. FASEB J. 2008;22(9):3186‐3195. 18511549 10.1096/fj.08-109181

[90] Reiman EM , Quiroz YT , Fleisher AS , et al. Brain imaging and fluid biomarker analysis in young adults at genetic risk for autosomal dominant Alzheimer's disease in the presenilin 1 E280A kindred: a case‐control study. Lancet Neurol. 2012;11(12):1048‐1056. 23137948 10.1016/S1474-4422(12)70228-4 PMC4181671

[91] Uemura K , Kuzuya A , Shimozono Y , et al. GSK3beta activity modifies the localization and function of presenilin 1. J Biol Chem. 2007;282(21):15823‐15832. 17389597 10.1074/jbc.M610708200

[92] Pigino G , Morfini G , Pelsman A , Mattson MP , Brady ST , Busciglio J . Alzheimer's presenilin 1 mutations impair kinesin‐based axonal transport. J Neurosci. 2003;23(11):4499‐4508. 12805290 10.1523/JNEUROSCI.23-11-04499.2003 PMC6740780

[93] Liu D , Wei N , Man HY , Lu Y , Zhu LQ , Wang JZ . The MT2 receptor stimulates axonogenesis and enhances synaptic transmission by activating Akt signaling. Cell Death Differ. 2015;22(4):583‐596. 25501601 10.1038/cdd.2014.195 PMC4356342

[94] Cao Q , Meng T , Man J , et al. aFGF promotes neurite growth by regulating GSK3beta‐CRMP2 signaling pathway in cortical neurons damaged by amyloid‐beta. J Alzheimers Dis. 2019;72(1):97‐109. 31561361 10.3233/JAD-190458

[95] Niehrs C . The complex world of WNT receptor signalling. Nat Rev Mol Cell Biol. 2012;13(12):767‐779. 23151663 10.1038/nrm3470

[96] Narvaes RF , Furini CRG . Role of Wnt signaling in synaptic plasticity and memory. Neurobiol Learn Mem. 2022;187:107558. 34808336 10.1016/j.nlm.2021.107558

[97] Taelman VF , Dobrowolski R , Plouhinec JL , et al. Wnt signaling requires sequestration of glycogen synthase kinase 3 inside multivesicular endosomes. Cell. 2010;143(7):1136‐1148. 21183076 10.1016/j.cell.2010.11.034 PMC3022472

[98] Folke J , Pakkenberg B , Brudek T . Impaired Wnt signaling in the prefrontal cortex of Alzheimer's disease. Mol Neurobiol. 2019;56(2):873‐891. 29804228 10.1007/s12035-018-1103-z

[99] Marzo A , Galli S , Lopes D , et al. Reversal of synapse degeneration by restoring Wnt signaling in the adult hippocampus. Curr Biol. 2016;26(19):2551‐2561. 27593374 10.1016/j.cub.2016.07.024 PMC5070786

[100] Farias GG , Godoy JA , Hernandez F , Avila J , Fisher A , Inestrosa NC . M1 muscarinic receptor activation protects neurons from beta‐amyloid toxicity. A role for Wnt signaling pathway. Neurobiol Dis. 2004;17(2):337‐348. 15474371 10.1016/j.nbd.2004.07.016

[101] Ma K , Yang LM , Chen HZ , Lu Y . Activation of muscarinic receptors inhibits glutamate‐induced GSK‐3beta overactivation in PC12 cells. Acta Pharmacol Sin. 2013;34(7):886‐892. 23685950 10.1038/aps.2013.42 PMC4002616

[102] Wegmann S , Biernat J , Mandelkow E . A current view on tau protein phosphorylation in Alzheimer's disease. Curr Opin Neurobiol. 2021;69:131‐138. 33892381 10.1016/j.conb.2021.03.003

[103] Goedert M , Spillantini MG , Jakes R , Rutherford D , Crowther RA . Multiple isoforms of human microtubule‐associated protein tau: sequences and localization in neurofibrillary tangles of Alzheimer's disease. Neuron. 1989;3(4):519‐526. 2484340 10.1016/0896-6273(89)90210-9

[104] Cleveland DW , Hwo SY , Kirschner MW . Physical and chemical properties of purified tau factor and the role of tau in microtubule assembly. J Mol Biol. 1977;116(2):227‐247. 146092 10.1016/0022-2836(77)90214-5

[105] Grundke‐Iqbal I , Iqbal K , Tung YC , Quinlan M , Wisniewski HM , Binder LI . Abnormal phosphorylation of the microtubule‐associated protein tau (tau) in Alzheimer cytoskeletal pathology. Proc Natl Acad Sci U S A. 1986;83(13):4913‐4917. 3088567 10.1073/pnas.83.13.4913 PMC323854

[106] Li S , Poon CH , Zhang Z , et al. MicroRNA‐128 suppresses tau phosphorylation and reduces amyloid‐beta accumulation by inhibiting the expression of GSK3beta, APPBP2, and mTOR in Alzheimer's disease. CNS Neurosci Ther. 2023;29(7):1848‐1864. 36880288 10.1111/cns.14143 PMC10324361

[107] Hanger DP , Anderton BH , Noble W . Tau phosphorylation: the therapeutic challenge for neurodegenerative disease. Trends Mol Med. 2009;15(3):112‐119. 19246243 10.1016/j.molmed.2009.01.003

[108] Rankin CA , Sun Q , Gamblin TC . Tau phosphorylation by GSK‐3beta promotes tangle‐like filament morphology. Mol Neurodegener. 2007;2:12. 17598919 10.1186/1750-1326-2-12 PMC1936422

[109] Zhou Q , Li S , Li M , et al. Human tau accumulation promotes glycogen synthase kinase‐3beta acetylation and thus upregulates the kinase: a vicious cycle in Alzheimer neurodegeneration. EBioMedicine. 2022;78:103970. 35339896 10.1016/j.ebiom.2022.103970 PMC8956943

[110] Huber CM , Yee C , May T , Dhanala A , Mitchell CS . Cognitive decline in preclinical Alzheimer's disease: amyloid‐Beta versus tauopathy. J Alzheimers Dis. 2018;61(1):265‐281. 29154274 10.3233/JAD-170490 PMC5734131

[111] Maurin H , Lechat B , Dewachter I , et al. Neurological characterization of mice deficient in GSK3alpha highlight pleiotropic physiological functions in cognition and pathological activity as tau kinase. Mol Brain. 2013;6:27. 23705847 10.1186/1756-6606-6-27 PMC3671145

[112] Cortes‐Gomez MA , Llorens‐Alvarez E , Alom J , et al. Tau phosphorylation by glycogen synthase kinase 3 beta modulates enzyme acetylcholinesterase expression. J Neurochem. 2021;157(6):2091‐2105. 32955735 10.1111/jnc.15189 PMC8359467

[113] Manning BD , Toker A . AKT/PKB signaling: navigating the network. Cell. 2017;169(3):381‐405. 28431241 10.1016/j.cell.2017.04.001 PMC5546324

[114] Guo H , Wang H , Wang C , et al. C‐reactive protein induces tau hyperphosphorylation via GSK3beta signaling pathway in SH‐SY5Y cells. J Mol Neurosci. 2015;56(2):519‐527. 25966642 10.1007/s12031-015-0572-z

[115] Sen T , Saha P , Jiang T , Sen N . Sulfhydration of AKT triggers tau‐phosphorylation by activating glycogen synthase kinase 3beta in Alzheimer's disease. Proc Natl Acad Sci U S A. 2020;117(8):4418‐4427. 32051249 10.1073/pnas.1916895117 PMC7049105

[116] Giovinazzo D , Bursac B , Sbodio JI , et al. Hydrogen sulfide is neuroprotective in Alzheimer's disease by sulfhydrating GSK3beta and inhibiting tau hyperphosphorylation. Proc Natl Acad Sci U S A. 2021;118(4):e2017225118. 33431651 10.1073/pnas.2017225118 PMC7848711

[117] Dunning CJ , McGauran G , Willen K , Gouras GK , O'Connell DJ , Linse S . Direct high affinity interaction between Abeta42 and GSK3alpha stimulates hyperphosphorylation of tau. A new molecular link in Alzheimer's disease? ACS Chem Nerosci. 2016;7(2):161‐170. 10.1021/acschemneuro.5b00262 PMC4759616 26618561

[118] Hein L . Adrenoceptors and signal transduction in neurons. Cell Tissue Res. 2006;326(2):541‐551. 16896948 10.1007/s00441-006-0285-2

[119] Cho JH , Johnson GV . Glycogen synthase kinase 3 beta induces caspase‐cleaved tau aggregation in situ. J Biol Chem. 2004;279(52):54716‐54723. 15494420 10.1074/jbc.M403364200

[120] Chu J , Lauretti E , Pratico D . Caspase‐3‐dependent cleavage of Akt modulates tau phosphorylation via GSK3beta kinase: implications for Alzheimer's disease. Mol Psychiatry. 2017;22(7):1002‐1008. 28138159 10.1038/mp.2016.214

[121] Moussaed M , Huc‐Brandt S , Cubedo N , et al. Regenerating islet‐derived 1alpha (REG‐1alpha) protein increases tau phosphorylation in cell and animal models of tauopathies. Neurobiol Dis. 2018;119:136‐148. 30092268 10.1016/j.nbd.2018.07.029

[122] Schubert M , Gautam D , Surjo D , et al. Role for neuronal insulin resistance in neurodegenerative diseases. Proc Natl Acad Sci U S A. 2004;101(9):3100‐3105. 14981233 10.1073/pnas.0308724101 PMC365750

[123] Wang WZ , Li MW , Chen Y , et al. 3xTg‐AD mice overexpressing phospholipid transfer protein improves cognition through decreasing amyloid‐beta production and tau hyperphosphorylation. J Alzheimers Dis. 2021;82(4):1635‐1649. 34219730 10.3233/JAD-210463

[124] Dong W , Albers JJ , Vuletic S . Phospholipid transfer protein reduces phosphorylation of tau in human neuronal cells. J Neurosci Res. 2009;87(14):3176‐3185. 19472218 10.1002/jnr.22137 PMC2755571

[125] Liu F , Gong X , Zhang G , Marquis K , Reinhart P , Andree TH . The inhibition of glycogen synthase kinase 3beta by a metabotropic glutamate receptor 5 mediated pathway confers neuroprotection to Abeta peptides. J Neurochem. 2005;95(5):1363‐1372. 16277616 10.1111/j.1471-4159.2005.03474.x

[126] Bloom GS . Amyloid‐beta and tau: the trigger and bullet in Alzheimer disease pathogenesis. JAMA Neurol. 2014;71(4):505‐508. 24493463 10.1001/jamaneurol.2013.5847 PMC12908160

[127] Zhang F , Gannon M , Chen Y , et al. Beta‐amyloid redirects norepinephrine signaling to activate the pathogenic GSK3beta/tau cascade. Sci Transl Med. 2020;12(526):eaay6931. 31941827 10.1126/scitranslmed.aay6931 PMC7891768

[128] Manassero G , Guglielmotto M , Zamfir R , et al. Beta‐amyloid 1‐42 monomers, but not oligomers, produce PHF‐like conformation of tau protein. Aging Cell. 2016;15(5):914‐923. 27406053 10.1111/acel.12500 PMC5013016

[129] Ma QL , Lim GP , Harris‐White ME , et al. Antibodies against beta‐amyloid reduce Abeta oligomers, glycogen synthase kinase‐3beta activation and tau phosphorylation in vivo and in vitro. J Neurosci Res. 2006;83(3):374‐384. 16385556 10.1002/jnr.20734

[130] Guo JP , Arai T , Miklossy J , McGeer PL . Abeta and tau form soluble complexes that may promote self aggregation of both into the insoluble forms observed in Alzheimer's disease. Proc Natl Acad Sci U S A. 2006;103(6):1953‐1958. 16446437 10.1073/pnas.0509386103 PMC1413647

[131] Park H , Kam TI , Kim Y , et al. Neuropathogenic role of adenylate kinase‐1 in Abeta‐mediated tau phosphorylation via AMPK and GSK3beta. Hum Mol Genet. 2012;21(12):2725‐2737. 22419736 10.1093/hmg/dds100

[132] Paquet C , Mouton‐Liger F , Meurs EF , et al. The PKR activator PACT is induced by Abeta: involvement in Alzheimer's disease. Brain Pathol. 2012;22(2):219‐229. 21790829 10.1111/j.1750-3639.2011.00520.x PMC8029131

[133] Zhang Y , Zhang Y , Aman Y , et al. Amyloid‐beta toxicity modulates tau phosphorylation through the PAX6 signalling pathway. Brain. 2021;144(9):2759‐2770. 34428276 10.1093/brain/awab134

[134] Chen KL , Yuan RY , Hu CJ , Hsu CY . Amyloid‐beta peptide alteration of tau exon‐10 splicing via the GSK3beta‐SC35 pathway. Neurobiol Dis. 2010;40(2):378‐385. 20615469 10.1016/j.nbd.2010.06.013

[135] Hernandez F , Perez M , Lucas JJ , Mata AM , Bhat R , Avila J . Glycogen synthase kinase‐3 plays a crucial role in tau exon 10 splicing and intranuclear distribution of SC35. Implications for Alzheimer's disease. J Biol Chem. 2004;279(5):3801‐3806. 14602710 10.1074/jbc.M311512200

[136] Harris SS , Wolf F , De Strooper B , Busche MA . Tipping the scales: peptide‐dependent dysregulation of neural circuit dynamics in Alzheimer's disease. Neuron. 2020;107(3):417‐435. 32579881 10.1016/j.neuron.2020.06.005

[137] Deng J , Habib A , Obregon DF , et al. Soluble amyloid precursor protein alpha inhibits tau phosphorylation through modulation of GSK3beta signaling pathway. J Neurochem. 2015;135(3):630‐637. 26342176 10.1111/jnc.13351 PMC4624213

[138] Zhao F , Wang C , Zhu X . Isoform‐specific roles of AMPK catalytic alpha subunits in Alzheimer's disease. J Clin Invest. 2020;130(7):3403‐3405. 32452835 10.1172/JCI137908 PMC7324169

[139] Wang L , Li N , Shi FX , et al. Upregulation of AMPK ameliorates Alzheimer's disease‐like tau pathology and memory impairment. Mol Neurobiol. 2020;57(8):3349‐3361. 32519244 10.1007/s12035-020-01955-w

[140] Wang L , Liu BJ , Cao Y , et al. Deletion of Type‐2 cannabinoid receptor induces Alzheimer's disease‐like tau pathology and memory impairment through AMPK/GSK3beta pathway. Mol Neurobiol. 2018;55(6):4731‐4744. 28717968 10.1007/s12035-017-0676-2

[141] Zhao S , Kusminski CM , Scherer PE . Adiponectin, leptin and cardiovascular disorders. Circ Res. 2021;128(1):136‐149. 33411633 10.1161/CIRCRESAHA.120.314458 PMC7799441

[142] Ng RC , Cheng OY , Jian M , et al. Chronic adiponectin deficiency leads to Alzheimer's disease‐like cognitive impairments and pathologies through AMPK inactivation and cerebral insulin resistance in aged mice. Mol Neurodegener. 2016;11(1):71. 27884163 10.1186/s13024-016-0136-x PMC5123368

[143] Lee CW , Shih YH , Wu SY , Yang T , Lin C , Kuo YM . Hypoglycemia induces tau hyperphosphorylation. Curr Alzheimer Res. 2013;10(3):298‐308. 23036024 10.2174/1567205011310030009

[144] Suo WZ . GRK5 deficiency causes mild cognitive impairment due to Alzheimer's disease. J Alzheimers Dis. 2022;85(4):1399‐1410. 34958040 10.3233/JAD-215379 PMC9413990

[145] Zhao J , Li X , Chen X , et al. GRK5 influences the phosphorylation of tau via GSK3beta and contributes to Alzheimer's disease. J Cell Physiol. 2019;234(7):10411‐10420. 30511419 10.1002/jcp.27709

[146] Wang S , Toth ME , Bereczki E , et al. Interplay between glycogen synthase kinase‐3beta and tau in the cerebellum of Hsp27 transgenic mouse. J Neurosci Res. 2011;89(8):1267‐1275. 21544852 10.1002/jnr.22660

[147] Pehar M , Ko MH , Li M , Scrable H , Puglielli L . P44, the ‘longevity‐assurance’ isoform of P53, regulates tau phosphorylation and is activated in an age‐dependent fashion. Aging Cell. 2014;13(3):449‐456. 24341977 10.1111/acel.12192 PMC4032616

[148] Cancino GI , Miller FD , Kaplan DR . p73 haploinsufficiency causes tau hyperphosphorylation and tau kinase dysregulation in mouse models of aging and Alzheimer's disease. Neurobiol Aging. 2013;34(2):387‐399. 22592019 10.1016/j.neurobiolaging.2012.04.010

[149] Greeve I , Hermans‐Borgmeyer I , Brellinger C , et al. The human DIMINUTO/DWARF1 homolog seladin‐1 confers resistance to Alzheimer's disease‐associated neurodegeneration and oxidative stress. J Neurosci. 2000;20(19):7345‐7352. 11007892 10.1523/JNEUROSCI.20-19-07345.2000 PMC6772756

[150] Qi Z , Zhang Y , Yao K , et al. DHCR24 knockdown lead to hyperphosphorylation of tau at Thr181, Thr231, Ser262, Ser396, and Ser422 sites by membrane lipid‐raft dependent PP2A signaling in SH‐SY5Y cells. Neurochem Res. 2021;46(7):1627‐1640. 33710538 10.1007/s11064-021-03273-6

[151] Roichman A , Elhanati S , Aon MA , et al. Restoration of energy homeostasis by SIRT6 extends healthy lifespan. Nat Commun. 2021;12(1):3208. 34050173 10.1038/s41467-021-23545-7 PMC8163764

[152] Kaluski S , Portillo M , Besnard A , et al. Neuroprotective functions for the histone deacetylase SIRT6. Cell Rep. 2017;18(13):3052‐3062. 28355558 10.1016/j.celrep.2017.03.008 PMC5389893

[153] Lloret A , Badia MC , Giraldo E , et al. Amyloid‐beta toxicity and tau hyperphosphorylation are linked via RCAN1 in Alzheimer's disease. J Alzheimers Dis. 2011;27(4):701‐709. 21876249 10.3233/JAD-2011-110890 PMC3690537

[154] Duan J , Marcellus KA , Qin X , Wang Y , Paudel HK . Cystatin C promotes tau protein phosphorylation and causes microtubule instability by inhibiting intracellular turnover of GSK3beta in neurons. Mol Cell Neurosci. 2018;89:1‐8. 29577984 10.1016/j.mcn.2018.03.009

[155] Wisely EV , Xiang YK , Oddo S . Genetic suppression of beta2‐adrenergic receptors ameliorates tau pathology in a mouse model of tauopathies. Hum Mol Genet. 2014;23(15):4024‐4034. 24626633 10.1093/hmg/ddu116 PMC4082366

[156] Cortes N , Guzman‐Martinez L , Andrade V , Gonzalez A , Maccioni RB . CDK5: A unique CDK and its multiple roles in the nervous system. J Alzheimers Dis. 2019;68(3):843‐855. 30856110 10.3233/JAD-180792

[157] Caccamo A , Oddo S , Billings LM , et al. M1 receptors play a central role in modulating AD‐like pathology in transgenic mice. Neuron. 2006;49(5):671‐682. 16504943 10.1016/j.neuron.2006.01.020

[158] Bitner RS , Nikkel AL , Markosyan S , Otte S , Puttfarcken P , Gopalakrishnan M . Selective alpha7 nicotinic acetylcholine receptor activation regulates glycogen synthase kinase3beta and decreases tau phosphorylation in vivo. Brain Res. 2009;1265:65‐74. 19230830 10.1016/j.brainres.2009.01.069

[159] Bitner RS , Markosyan S , Nikkel AL , Brioni JD . In‐vivo histamine H3 receptor antagonism activates cellular signaling suggestive of symptomatic and disease modifying efficacy in Alzheimer's disease. Neuropharmacology. 2011;60(2–3):460‐466. 21044639 10.1016/j.neuropharm.2010.10.026

[160] Hu J , Yang Y , Wang M , et al. Complement C3a receptor antagonist attenuates tau hyperphosphorylation via glycogen synthase kinase 3beta signaling pathways. Eur J Pharmacol. 2019;850:135‐140. 30771350 10.1016/j.ejphar.2019.02.020

[161] Bridi MS , Hawk JD , Chatterjee S , Safe S , Abel T . Pharmacological activators of the NR4A nuclear receptors enhance LTP in a CREB/CBP‐dependent manner. Neuropsychopharmacology. 2017;42(6):1243‐1253. 27834392 10.1038/npp.2016.253 PMC5437882

[162] Zhao LG , Tang Y , Tan JZ , Wang JW , Chen GJ , Zhu BL . The effect of NR4A1 on APP metabolism and tau phosphorylation. Genes Dis. 2018;5(4):342‐348. 30591936 10.1016/j.gendis.2018.04.008 PMC6304284

[163] Bergemalm D , Andersson E , Hultdin J , et al. Systemic inflammation in preclinical ulcerative colitis. Gastroenterology. 2021;161(5):1526‐1539.e9. 34298022 10.1053/j.gastro.2021.07.026

[164] Zhu C , Xu B , Sun X , Zhu Q , Sui Y . Targeting CCR3 to reduce amyloid‐beta production, tau hyperphosphorylation, and synaptic loss in a mouse model of Alzheimer's disease. Mol Neurobiol. 2017;54(10):7964‐7978. 27878757 10.1007/s12035-016-0269-5

[165] Takashima A , Murayama M , Murayama O , et al. Presenilin 1 associates with glycogen synthase kinase‐3beta and its substrate tau. Proc Natl Acad Sci U S A. 1998;95(16):9637‐9641. 9689133 10.1073/pnas.95.16.9637 PMC21391

[166] Chow HM , Guo D , Zhou JC , et al. CDK5 activator protein p25 preferentially binds and activates GSK3beta. Proc Natl Acad Sci U S A. 2014;111(45):E4887‐E4895. 25331900 10.1073/pnas.1402627111 PMC4234543

[167] Lu B , Al‐Ramahi I , Valencia A , et al. Identification of NUB1 as a suppressor of mutant Huntington toxicity via enhanced protein clearance. Nat Neurosci. 2013;16(5):562‐570. 23525043 10.1038/nn.3367

[168] Richet E , Pooler AM , Rodriguez T , et al. NUB1 modulation of GSK3beta reduces tau aggregation. Hum Mol Genet. 2012;21(24):5254‐5267. 22965877 10.1093/hmg/dds376

[169] Stoothoff WH , Bailey CD , Mi K , Lin SC , Johnson GV . Axin negatively affects tau phosphorylation by glycogen synthase kinase 3beta. J Neurochem. 2002;83(4):904‐913. 12421363 10.1046/j.1471-4159.2002.01197.x

[170] Sharpe AH , Pauken KE . The diverse functions of the PD1 inhibitory pathway. Nat Rev Immunol. 2018;18(3):153‐167. 28990585 10.1038/nri.2017.108

[171] Zou Y , Gan CL , Xin Z , et al. Programmed cell death protein 1 blockade reduces glycogen synthase kinase 3beta activity and tau hyperphosphorylation in Alzheimer's disease mouse models. Front Cell Dev Biol. 2021;9:769229. 34977020 10.3389/fcell.2021.769229 PMC8716757

[172] Ishii T , Furuoka H , Muroi Y , Nishimura M . Inactivation of integrin‐linked kinase induces aberrant tau phosphorylation via sustained activation of glycogen synthase kinase 3beta in N1E‐115 neuroblastoma cells. J Biol Chem. 2003;278(29):26970‐26975. 12714590 10.1074/jbc.M304113200

[173] Salcedo‐Tello P , Hernandez‐Ortega K , Arias C . Susceptibility to GSK3beta‐induced tau phosphorylation differs between the young and aged hippocampus after Wnt signaling inhibition. J Alzheimers Dis. 2014;39(4):775‐785. 24270208 10.3233/JAD-130749

[174] Koikawa K , Kibe S , Suizu F , et al. Targeting Pin1 renders pancreatic cancer eradicable by synergizing with immunochemotherapy. Cell. 2021;184(18):4753‐4771.e27. 34388391 10.1016/j.cell.2021.07.020 PMC8557351

[175] Xiong YS , Wang DL , Tan L , et al. Inhibition of glycogen synthase kinase‐3 reverses tau hyperphosphorylation induced by Pin1 down‐regulation. CNS Neurol Disord Drug Targets. 2013;12(3):436‐443. 23469846 10.2174/1871527311312030016

[176] Lidon L , Llao‐Hierro L , Nuvolone M , et al. Tau exon 10 inclusion by PrP(C) through downregulating GSK3beta activity. Int J Mol Sci. 2021;22(10):5370. 34065232 10.3390/ijms22105370 PMC8161268

[177] Li A , Ceballos‐Diaz C , DiNunno N , et al. IFN‐gamma promotes tau phosphorylation without affecting mature tangles. FASEB J. 2015;29(10):4384‐4398. 26156074 10.1096/fj.15-275834 PMC6137542

[178] Li J , Chen W , Yi Y , Tong Q . miR‐219‐5p inhibits tau phosphorylation by targeting TTBK1 and GSK‐3beta in Alzheimer's disease. J Cell Biochem. 2019;120(6):9936‐9946. 30556160 10.1002/jcb.28276

[179] El Fatimy R , Li S , Chen Z , et al. MicroRNA‐132 provides neuroprotection for tauopathies via multiple signaling pathways. Acta Neuropathol. 2018;136(4):537‐555. 29982852 10.1007/s00401-018-1880-5 PMC6132948

[180] Pichler S , Gu W , Hartl D , et al. The miRNome of Alzheimer's disease: consistent downregulation of the miR‐132/212 cluster. Neurobiol Aging. 2017;50:167.e1‐167.e10. 10.1016/j.neurobiolaging.2016.09.019 27816213

[181] Patrick E , Rajagopal S , Wong HA , et al. Dissecting the role of non‐coding RNAs in the accumulation of amyloid and tau neuropathologies in Alzheimer's disease. Mol Neurodegener. 2017;12(1):51. 28668092 10.1186/s13024-017-0191-y PMC5494142

[182] Jiang H , Liu J , Guo S , et al. miR‐23b‐3p rescues cognition in Alzheimer's disease by reducing tau phosphorylation and apoptosis via GSK‐3beta signaling pathways. Mol Ther Nucleic Acids. 2022;28:539‐557. 35592504 10.1016/j.omtn.2022.04.008 PMC9092887

[183] Zhao Y , Wang Z , Mao Y , et al. NEAT1 regulates microtubule stabilization via FZD3/GSK3beta/P‐tau pathway in SH‐SY5Y cells and APP/PS1 mice. Aging. 2020;12(22):23233‐23250. 33221742 10.18632/aging.104098 PMC7746375

[184] Kandimalla R , Thirumala V , Reddy PH . Is Alzheimer's disease a type 3 diabetes? A critical appraisal. Biochim Biophys Acta Mol Basis Dis. 2017;1863(5):1078‐1089. 27567931 10.1016/j.bbadis.2016.08.018 PMC5344773

[185] Dey A , Hao S , Wosiski‐Kuhn M , Stranahan AM . Glucocorticoid‐mediated activation of GSK3beta promotes tau phosphorylation and impairs memory in type 2 diabetes. Neurobiol Aging. 2017;57:75‐83. 28609678 10.1016/j.neurobiolaging.2017.05.010 PMC5534373

[186] Wang X , Zheng W , Xie JW , et al. Insulin deficiency exacerbates cerebral amyloidosis and behavioral deficits in an Alzheimer transgenic mouse model. Mol Neurodegener. 2010;5:46. 21044348 10.1186/1750-1326-5-46 PMC2987993

[187] Crunfli F , Mazucanti CH , de Moraes RCM , et al. NO‐dependent Akt inactivation by S‐nitrosylation as a possible mechanism of STZ‐induced neuronal insulin resistance. J Alzheimers Dis. 2018;65(4):1427‐1443. 30149447 10.3233/JAD-180284

[188] Hongo H , Kihara T , Kume T , et al. Glycogen synthase kinase‐3beta activation mediates rotenone‐induced cytotoxicity with the involvement of microtubule destabilization. Biochem Biophys Res Commun. 2012;426(1):94‐99. 22922102 10.1016/j.bbrc.2012.08.042

[189] Xu H , Chen X , Wang J , et al. Involvement of insulin signalling pathway in methamphetamine‐induced hyperphosphorylation of tau. Toxicology. 2018;408:88‐94. 29981415 10.1016/j.tox.2018.07.002

[190] Maurya SK , Mishra J , Abbas S , Bandyopadhyay S . Cypermethrin stimulates GSK3beta‐dependent Abeta and p‐tau proteins and cognitive loss in young rats: reduced HB‐EGF signaling and downstream neuroinflammation as critical regulators. Mol Neurobiol. 2016;53(2):968‐982. 25575682 10.1007/s12035-014-9061-6

[191] Lahiani‐Cohen I , Touloumi O , Lagoudaki R , Grigoriadis N , Rosenmann H . Exposure to 3‐nitropropionic acid mitochondrial toxin induces tau pathology in tangle‐mouse model and in wild type‐mice. Front Cell Dev Biol. 2019;7:321. 32010684 10.3389/fcell.2019.00321 PMC6971403

[192] Wisessaowapak C , Visitnonthachai D , Watcharasit P , Satayavivad J . Prolonged arsenic exposure increases tau phosphorylation in differentiated SH‐SY5Y cells: the contribution of GSK3 and ERK1/2. Environ Toxicol Pharmacol. 2021;84:103626. 33621689 10.1016/j.etap.2021.103626

[193] Gassowska M , Baranowska‐Bosiacka I , Moczydlowska J , et al. Perinatal exposure to lead (Pb) promotes tau phosphorylation in the rat brain in a GSK‐3beta and CDK5 dependent manner: relevance to neurological disorders. Toxicology. 2016;347‐349:17‐28. 10.1016/j.tox.2016.03.002 27012722

[194] Guo C , Wang P , Zhong ML , et al. Deferoxamine inhibits iron induced hippocampal tau phosphorylation in the Alzheimer transgenic mouse brain. Neurochem Int. 2013;62(2):165‐172. 23262393 10.1016/j.neuint.2012.12.005

[195] Wu TY , Zhao LX , Zhang YH , Fan YG . Activation of vitamin D receptor inhibits tau phosphorylation is associated with reduction of iron accumulation in APP/PS1 transgenic mice. Neurochem Int. 2022;153:105260. 34953963 10.1016/j.neuint.2021.105260

[196] Cui B , Zhu L , She X , et al. Chronic noise exposure causes persistence of tau hyperphosphorylation and formation of NFT tau in the rat hippocampus and prefrontal cortex. Exp Neurol. 2012;238(2):122‐129. 22971273 10.1016/j.expneurol.2012.08.028

[197] Nicolia V , Fuso A , Cavallaro RA , Di Luzio A , Scarpa S . B vitamin deficiency promotes tau phosphorylation through regulation of GSK3beta and PP2A. J Alzheimers Dis. 2010;19(3):895‐907. 20157245 10.3233/JAD-2010-1284

[198] Fujio J , Hosono H , Ishiguro K , Ikegami S , Fujita SC . Tau phosphorylation in the mouse brain during aversive conditioning. Neurochem Int. 2007;51(2–4):200‐208. 17597257 10.1016/j.neuint.2007.04.024

[199] Ikeda Y , Ishiguro K , Fujita SC . Ether stress‐induced Alzheimer‐like tau phosphorylation in the normal mouse brain. FEBS Lett. 2007;581(5):891‐897. 17289030 10.1016/j.febslet.2007.01.064

[200] Chen GF , Xu TH , Yan Y , et al. Amyloid beta: structure, biology and structure‐based therapeutic development. Acta Pharmacol Sin. 2017;38(9):1205‐1235. 28713158 10.1038/aps.2017.28 PMC5589967

[201] Guerreiro RJ , Gustafson DR , Hardy J . The genetic architecture of Alzheimer's disease: beyond APP, PSENs and APOE. Neurobiol Aging. 2012;33(3):437‐456. 20594621 10.1016/j.neurobiolaging.2010.03.025 PMC2980860

[202] Haass C , Lemere CA , Capell A , et al. The Swedish mutation causes early‐onset Alzheimer's disease by beta‐secretase cleavage within the secretory pathway. Nat Med. 1995;1(12):1291‐1296. 7489411 10.1038/nm1295-1291

[203] Castro‐Alvarez JF , Uribe‐Arias A , Cardona‐Gomez GP . Cyclin‐dependent kinase 5 targeting prevents beta‐amyloid aggregation involving glycogen synthase kinase 3beta and phosphatases. J Neurosci Res. 2015;93(8):1258‐1266. 25711385 10.1002/jnr.23576 PMC4478163

[204] Gupta S , Singh V , Ganesh S , Singhal NK , Sandhir R . siRNA mediated GSK3beta knockdown targets insulin signaling pathway and rescues Alzheimer's disease pathology: evidence from in vitro and in vivo studies. ACS Appl Mater Interfaces. 2022;14(1):69‐93. 34967205 10.1021/acsami.1c15305

[205] Israel MA , Yuan SH , Bardy C , et al. Probing sporadic and familial Alzheimer's disease using induced pluripotent stem cells. Nature. 2012;482(7384):216‐220. 22278060 10.1038/nature10821 PMC3338985

[206] Chen L , Xu S , Wu T , et al. Abnormal platelet amyloid‐beta precursor protein metabolism in SAMP8 mice: evidence for peripheral marker in Alzheimer's disease. J Cell Physiol. 2019;234(12):23528‐23536. 31183859 10.1002/jcp.28921

[207] Fang F , Yu Q , Arancio O , et al. RAGE mediates Abeta accumulation in a mouse model of Alzheimer's disease via modulation of beta‐ and gamma‐secretase activity. Hum Mol Genet. 2018;27(6):1002‐1014. 29329433 10.1093/hmg/ddy017 PMC6075512

[208] Parr C , Mirzaei N , Christian M , Sastre M . Activation of the Wnt/beta‐catenin pathway represses the transcription of the beta‐amyloid precursor protein cleaving enzyme (BACE1) via binding of T‐cell factor‐4 to BACE1 promoter. FASEB J. 2015;29(2):623‐635. 25384422 10.1096/fj.14-253211

[209] Hohman TJ , Chibnik L , Bush WS , et al. GSK3beta interactions with amyloid genes: An autopsy verification and extension. Neurotox Res. 2015;28(3):232‐238. 26194614 10.1007/s12640-015-9541-0 PMC4625986

[210] Readhead B , Haure‐Mirande JV , Funk CC , et al. Multiscale analysis of independent Alzheimer's cohorts finds disruption of molecular, genetic, and clinical networks by human herpesvirus. Neuron. 2018;99(1):64‐82.e7. 29937276 10.1016/j.neuron.2018.05.023 PMC6551233

[211] Li Z , Moniruzzaman M , Dastgheyb RM , et al. Astrocytes deliver CK1 to neurons via extracellular vesicles in response to inflammation promoting the translation and amyloidogenic processing of APP. J Extracell Vesicles. 2020;10(2):e12035. 33408815 10.1002/jev2.12035 PMC7775567

[212] Rockenstein E , Torrance M , Adame A , et al. Neuroprotective effects of regulators of the glycogen synthase kinase‐3beta signaling pathway in a transgenic model of Alzheimer's disease are associated with reduced amyloid precursor protein phosphorylation. J Neurosci. 2007;27(8):1981‐1991. 17314294 10.1523/JNEUROSCI.4321-06.2007 PMC6673566

[213] Triaca V , Sposato V , Bolasco G , et al. NGF controls APP cleavage by downregulating APP phosphorylation at Thr668: relevance for Alzheimer's disease. Aging Cell. 2016;15(4):661‐672. 27076121 10.1111/acel.12473 PMC4933663

[214] Parr C , Carzaniga R , Gentleman SM , Van Leuven F , Walter J , Sastre M . Glycogen synthase kinase 3 inhibition promotes lysosomal biogenesis and autophagic degradation of the amyloid‐beta precursor protein. Mol Cell Biol. 2012;32(21):4410‐4418. 22927642 10.1128/MCB.00930-12 PMC3486153

[215] Isla AG , Vazquez‐Cuevas FG , Pena‐Ortega F . Exercise prevents amyloid‐beta‐induced hippocampal network disruption by inhibiting GSK3beta activation. J Alzheimers Dis. 2016;52(1):333‐343. 27003207 10.3233/JAD-150352

[216] Cummings J , Lee G , Nahed P , et al. Alzheimer's disease drug development pipeline: 2022. Alzheimers Dement. 2022;8(1):e12295. 10.1002/trc2.12295 PMC9066743 35516416

[217] van Olst L , Kamermans A , Halters S , et al. Adaptive immune changes associate with clinical progression of Alzheimer's disease. Mol Neurodegener. 2024;19(1):38. 38658964 10.1186/s13024-024-00726-8 PMC11044380

[218] Silva‐Palacios A , Ostolga‐Chavarria M , Zazueta C , Konigsberg M . Nrf2: molecular and epigenetic regulation during aging. Ageing Res Rev. 2018;47:31‐40. 29913211 10.1016/j.arr.2018.06.003

[219] Xiong W , Liu Y , Zhou H , et al. Human dental pulp stem cells mitigate the neuropathology and cognitive decline via AKT‐GSK3beta‐Nrf2 pathways in Alzheimer's disease. Int J Oral Sci. 2024;16(1):40. 38740746 10.1038/s41368-024-00300-4 PMC11091120

[220] George M , Tharakan M , Culberson J , Reddy AP , Reddy PH . Role of Nrf2 in aging, Alzheimer's and other neurodegenerative diseases. Ageing Res Rev. 2022;82:101756. 36243357 10.1016/j.arr.2022.101756

[221] Farr SA , Ripley JL , Sultana R , et al. Antisense oligonucleotide against GSK‐3beta in brain of SAMP8 mice improves learning and memory and decreases oxidative stress: involvement of transcription factor Nrf2 and implications for Alzheimer disease. Free Radic Biol Med. 2014;67:387‐395. 24355211 10.1016/j.freeradbiomed.2013.11.014 PMC3945161

[222] Espada S , Rojo AI , Salinas M , Cuadrado A . The muscarinic M1 receptor activates Nrf2 through a signaling cascade that involves protein kinase C and inhibition of GSK‐3beta: connecting neurotransmission with neuroprotection. J Neurochem. 2009;110(3):1107‐1119. 19558456 10.1111/j.1471-4159.2009.06208.x

[223] Zhou Y , Men L , Sun Y , Wei M , Fan X . Pharmacodynamic effects and molecular mechanisms of lignans from Schisandra chinensis Turcz. (Baill.), a current review. Eur J Pharmacol. 2021;892:173796. 33345853 10.1016/j.ejphar.2020.173796

[224] Chou CH , Hsu KC , Lin TE , Yang CR . Anti‐inflammatory and tau phosphorylation‐inhibitory effects of Eupatin. Molecules. 2020;25(23):5652. 33266202 10.3390/molecules25235652 PMC7731404

[225] Wei T , Wang Y , Xu W , Liu Y , Chen H , Yu Z . KCa3.1 deficiency attenuates neuroinflammation by regulating an astrocyte phenotype switch involving the PI3K/AKT/GSK3beta pathway. Neurobiol Dis. 2019;132:104588. 31470105 10.1016/j.nbd.2019.104588

[226] Wagner KM , McReynolds CB , Schmidt WK , Hammock BD . Soluble epoxide hydrolase as a therapeutic target for pain, inflammatory and neurodegenerative diseases. Pharmacol Ther. 2017;180:62‐76. 28642117 10.1016/j.pharmthera.2017.06.006 PMC5677555

[227] Sun CP , Zhang XY , Zhou JJ , et al. Inhibition of sEH via stabilizing the level of EETs alleviated Alzheimer's disease through GSK3beta signaling pathway. Food Chem Toxicol. 2021;156:112516. 34411643 10.1016/j.fct.2021.112516 PMC8889936

[228] Chong ZZ , Li F , Maiese K . Cellular demise and inflammatory microglial activation during beta‐amyloid toxicity are governed by Wnt1 and canonical signaling pathways. Cell Signal. 2007;19(6):1150‐1162. 17289346 10.1016/j.cellsig.2006.12.009 PMC1913492

[229] Zu HB , Liu XY , Yao K . DHCR24 overexpression modulates microglia polarization and inflammatory response via Akt/GSK3beta signaling in Abeta25‐35 treated BV‐2 cells. Life Sci. 2020;260:118470. 32950573 10.1016/j.lfs.2020.118470

[230] Ko CY , Wang WL , Wang SM , Chu YY , Chang WC , Wang JM . Glycogen synthase kinase‐3beta‐mediated CCAAT/enhancer‐binding protein delta phosphorylation in astrocytes promotes migration and activation of microglia/macrophages. Neurobiol Aging. 2014;35(1):24‐34. 23993701 10.1016/j.neurobiolaging.2013.07.021

[231] Petry FDS , Coelho BP , Gaelzer MM , et al. Genistein protects against amyloid‐beta‐induced toxicity in SH‐SY5Y cells by regulation of Akt and tau phosphorylation. Phytother Res. 2020;34(4):796‐807. 31795012 10.1002/ptr.6560

[232] Green HF , Nolan YM . GSK‐3 mediates the release of IL‐1beta, TNF‐alpha and IL‐10 from cortical glia. Neurochem Int. 2012;61(5):666‐671. 22796213 10.1016/j.neuint.2012.07.003

[233] Ashleigh T , Swerdlow RH , Beal MF . The role of mitochondrial dysfunction in Alzheimer's disease pathogenesis. Alzheimers Dement. 2023;19(1):333‐342. 35522844 10.1002/alz.12683

[234] Tanno M , Kuno A , Ishikawa S , et al. Translocation of glycogen synthase kinase‐3beta (GSK‐3beta), a trigger of permeability transition, is kinase activity‐dependent and mediated by interaction with voltage‐dependent anion channel 2 (VDAC2). J Biol Chem. 2014;289(42):29285‐29296. 25187518 10.1074/jbc.M114.563924 PMC4200279

[235] Martin SA , Souder DC , Miller KN , et al. GSK3beta regulates brain energy metabolism. Cell Rep. 2018;23(7):1922‐1931 e4. 29768193 10.1016/j.celrep.2018.04.045 PMC6082412

[236] Yan J , Liu XH , Han MZ , et al. Blockage of GSK3beta‐mediated Drp1 phosphorylation provides neuroprotection in neuronal and mouse models of Alzheimer's disease. Neurobiol Aging. 2015;36(1):211‐227. 25192600 10.1016/j.neurobiolaging.2014.08.005

[237] Rui Y , Tiwari P , Xie Z , Zheng JQ . Acute impairment of mitochondrial trafficking by beta‐amyloid peptides in hippocampal neurons. J Neurosci. 2006;26(41):10480‐10487. 17035532 10.1523/JNEUROSCI.3231-06.2006 PMC6674697

[238] Chen S , Owens GC , Makarenkova H , Edelman DB . HDAC6 regulates mitochondrial transport in hippocampal neurons. PLoS One. 2010;5(5):e10848. 20520769 10.1371/journal.pone.0010848 PMC2877100

[239] Davoody S , Asgari Taei A , Khodabakhsh P , Dargahi L . mTOR signaling and Alzheimer's disease: what we know and where we are? CNS Neurosci Ther. 2024;30(4):e14463. 37721413 10.1111/cns.14463 PMC11017461

[240] Bai X , Wu J , Zhang M , et al. DHCR24 knock‐down induced tau hyperphosphorylation at Thr181, Ser199, Thr231, Ser262, Ser396 epitopes and inhibition of autophagy by overactivation of GSK3beta/mTOR signaling. Front Aging Neurosci. 2021;13:513605. 33967735 10.3389/fnagi.2021.513605 PMC8098657

[241] Chong CM , Ke M , Tan Y , et al. Presenilin 1 deficiency suppresses autophagy in human neural stem cells through reducing gamma‐secretase‐independent ERK/CREB signaling. Cell Death Dis. 2018;9(9):879. 30158533 10.1038/s41419-018-0945-7 PMC6115391

[242] Meijer L , Flajolet M , Greengard P . Pharmacological inhibitors of glycogen synthase kinase 3. Trends Pharmacol Sci. 2004;25(9):471‐480. 15559249 10.1016/j.tips.2004.07.006

[243] Bhat R , Xue Y , Berg S , et al. Structural insights and biological effects of glycogen synthase kinase 3‐specific inhibitor AR‐A014418. J Biol Chem. 2003;278(46):45937‐45945. 12928438 10.1074/jbc.M306268200

[244] Amaral B , Capacci A , Anderson T , et al. Elucidation of the GSK3alpha structure informs the design of novel, paralog‐selective inhibitors. ACS Chem Nerosci. 2023;14(6):1080‐1094. 10.1021/acschemneuro.2c00476 PMC10020971 36812145

[245] Hartz RA , Ahuja VT , Sivaprakasam P , et al. Design, structure‐activity relationships, and in vivo evaluation of potent and brain‐penetrant Imidazo[1,2‐b]pyridazines as glycogen synthase kinase‐3beta (GSK‐3beta) inhibitors. J Med Chem. 2023;66(6):4231‐4252. 36950863 10.1021/acs.jmedchem.3c00133

[246] Dong Y , Lu J , Zhang S , et al. Design, synthesis and bioevaluation of 1,2,4‐thiadiazolidine‐3,5‐dione derivatives as potential GSK‐3beta inhibitors for the treatment of Alzheimer's disease. Bioorg Chem. 2023;134:106446. 36868127 10.1016/j.bioorg.2023.106446

[247] Jiang X , Liu C , Zou M , et al. Discovery of 2‐(cyclopropanecarboxamido)‐N‐(5‐((1‐(4‐fluorobenzyl)piperidin‐4‐yl)methoxy)pyridin‐3‐yl)isonicotinamide as a potent dual AChE/GSK3beta inhibitor for the treatment of Alzheimer's disease: significantly increasing the level of acetylcholine in the brain without affecting that in intestine. Eur J Med Chem. 2021;223:113663. 34198150 10.1016/j.ejmech.2021.113663

[248] Luo G , Chen L , Burton CR , et al. Discovery of isonicotinamides as highly selective, brain penetrable, and orally active glycogen synthase kinase‐3 inhibitors. J Med Chem. 2016;59(3):1041‐1051. 26751161 10.1021/acs.jmedchem.5b01550

[249] Reinhardt L , Kordes S , Reinhardt P , et al. Dual inhibition of GSK3beta and CDK5 protects the cytoskeleton of neurons from neuroinflammatory‐mediated degeneration in vitro and in vivo. Stem Cell Rep. 2019;12(3):502‐517. 10.1016/j.stemcr.2019.01.015 PMC6409486 30773488

[250] Jiang X , Zhou J , Wang Y , et al. Rational design and biological evaluation of a new class of thiazolopyridyl tetrahydroacridines as cholinesterase and GSK‐3 dual inhibitors for Alzheimer's disease. Eur J Med Chem. 2020;207:112751. 32950908 10.1016/j.ejmech.2020.112751

[251] Prati F , De Simone A , Armirotti A , et al. 3,4‐Dihydro‐1,3,5‐triazin‐2(1H)‐ones as the first dual BACE‐1/GSK‐3beta fragment hits against Alzheimer's disease. ACS Chem Nerosci. 2015;6(10):1665‐1682. 10.1021/acschemneuro.5b00121 26171616

[252] Saitoh M , Kunitomo J , Kimura E , et al. 2‐3‐[4‐(Alkylsulfinyl)phenyl]‐1‐benzofuran‐5‐yl‐5‐methyl‐1,3,4‐oxadiazole derivatives as novel inhibitors of glycogen synthase kinase‐3beta with good brain permeability. J Med Chem. 2009;52(20):6270‐6286. 19775160 10.1021/jm900647e

[253] Qu L , Li S , Ji L , et al. Discovery of PT‐65 as a highly potent and selective proteolysis‐targeting chimera degrader of GSK3 for treating Alzheimer's disease. Eur J Med Chem. 2021;226:113889. 34649182 10.1016/j.ejmech.2021.113889

[254] Liu JG , Zhao D , Gong Q , et al. Development of bisindole‐substituted aminopyrazoles as novel GSK‐3beta inhibitors with suppressive effects against microglial inflammation and oxidative neurotoxicity. ACS Chem Nerosci. 2020;11(20):3398‐3408. 10.1021/acschemneuro.0c00520 32960565

[255] Zheng R , Zhang ZH , Chen C , et al. Selenomethionine promoted hippocampal neurogenesis via the PI3K‐Akt‐GSK3beta‐Wnt pathway in a mouse model of Alzheimer's disease. Biochem Biophys Res Commun. 2017;485(1):6‐15. 28109879 10.1016/j.bbrc.2017.01.069

[256] Lin K , Sze SC , Liu B , et al. 20(S)‐protopanaxadiol and oleanolic acid ameliorate cognitive deficits in APP/PS1 transgenic mice by enhancing hippocampal neurogenesis. J Ginseng Res. 2021;45(2):325‐333. 33841013 10.1016/j.jgr.2020.07.003 PMC8020272

[257] Xiao HH , Zhang MB , Xu JT , et al. Icarisid II promotes proliferation and neuronal differentiation of neural stem cells via activating Wnt/beta‐catenin signaling pathway. Phytother Res. 2021;35:2773‐2784. 33455039 10.1002/ptr.7022

[258] Xiao HH , Chen JC , Li H , et al. Icarisid II rescues cognitive dysfunction via activation of Wnt/beta‐catenin signaling pathway promoting hippocampal neurogenesis in APP/PS1 transgenic mice. Phytother Res. 2022;36(5):2095‐2108. 35230733 10.1002/ptr.7430

[259] Gong EJ , Park HR , Kim ME , et al. Morin attenuates tau hyperphosphorylation by inhibiting GSK3beta. Neurobiol Dis. 2011;44(2):223‐230. 21782947 10.1016/j.nbd.2011.07.005 PMC3166962

[260] Kim K , Cha JS , Kim JS , Ahn J , Ha NC , Cho HS . Crystal structure of GSK3beta in complex with the flavonoid, morin. Biochem Biophys Res Commun. 2018;504(2):519‐524. 30197003 10.1016/j.bbrc.2018.08.182

[261] Song XY , Hu JF , Chu SF , et al. Ginsenoside Rg1 attenuates okadaic acid induced spatial memory impairment by the GSK3beta/tau signaling pathway and the Abeta formation prevention in rats. Eur J Pharmacol. 2013;710(1–3):29‐38. 23588117 10.1016/j.ejphar.2013.03.051

[262] Zhao HH , Di J , Liu WS , Liu HL , Lai H , Lu YL . Involvement of GSK3 and PP2A in ginsenoside Rb1's attenuation of aluminum‐induced tau hyperphosphorylation. Behav Brain Res. 2013;241:228‐234. 23219964 10.1016/j.bbr.2012.11.037

[263] Yang Y , Wang L , Zhang C , et al. Ginsenoside Rg1 improves Alzheimer's disease by regulating oxidative stress, apoptosis, and neuroinflammation through Wnt/GSK‐3beta/beta‐catenin signaling pathway. Chem Biol Drug Des. 2022;99(6):884‐896. 35313087 10.1111/cbdd.14041

[264] Li L , Liu Z , Liu J , et al. Ginsenoside Rd attenuates beta‐amyloid‐induced tau phosphorylation by altering the functional balance of glycogen synthase kinase 3beta and protein phosphatase 2A. Neurobiol Dis. 2013;54:320‐328. 23321003 10.1016/j.nbd.2013.01.002

[265] Hu XL , Guo C , Hou JQ , et al. Stereoisomers of schisandrin B are potent ATP competitive GSK‐3beta inhibitors with neuroprotective effects against Alzheimer's disease: stereochemistry and biological activity. ACS Chem Nerosci. 2019;10(2):996‐1007. 10.1021/acschemneuro.8b00252 29944335

[266] Xu M , Dong Y , Wan S , et al. Schisantherin B ameliorates Abeta1‐42‐induced cognitive decline via restoration of GLT‐1 in a mouse model of Alzheimer's disease. Physiol Behav. 2016;167:265‐273. 27660034 10.1016/j.physbeh.2016.09.018

[267] Ma XH , Duan WJ , Mo YS , et al. Neuroprotective effect of paeoniflorin on okadaic acid‐induced tau hyperphosphorylation via calpain/Akt/GSK‐3beta pathway in SH‐SY5Y cells. Brain Res. 2018;1690:1‐11. 29596798 10.1016/j.brainres.2018.03.022

[268] Wu Y , Chen Q , Wen B , Wu N , He B , Chen J . Berberine reduces Abeta42 deposition and tau hyperphosphorylation via ameliorating endoplasmic reticulum stress. Front Pharmacol. 2021;12:640758. 34349640 10.3389/fphar.2021.640758 PMC8327086

[269] Xiao S , Wu Q , Yao X , et al. Inhibitory effects of isobavachalcone on tau protein aggregation, tau phosphorylation, and oligomeric tau‐induced apoptosis. ACS Chem Nerosci. 2021;12(1):123‐132. 10.1021/acschemneuro.0c00617 33320518

[270] Huang JM , Huang FI , Yang CR . Moscatilin ameliorates tau phosphorylation and cognitive deficits in Alzheimer's disease models. J Nat Prod. 2019;82(7):1979‐1988. 31291099 10.1021/acs.jnatprod.9b00375

[271] Huang X , Wang J , Chen X , et al. The prenylflavonoid xanthohumol reduces Alzheimer‐like changes and modulates multiple pathogenic molecular pathways in the Neuro2a/APPswe cell model of AD. Front Pharmacol. 2018;9:199. 29670521 10.3389/fphar.2018.00199 PMC5893754

[272] Jhang KA , Park JS , Kim HS , Chong YH . Resveratrol ameliorates tau hyperphosphorylation at Ser396 site and oxidative damage in rat hippocampal slices exposed to vanadate: implication of ERK1/2 and GSK‐3beta signaling cascades. J Agric Food Chem. 2017;65(44):9626‐9634. 29022339 10.1021/acs.jafc.7b03252

[273] Ahmad A , Ali T , Park HY , Badshah H , Rehman SU , Kim MO . Neuroprotective effect of Fisetin against amyloid‐Beta‐induced cognitive/synaptic dysfunction, neuroinflammation, and neurodegeneration in adult mice. Mol Neurobiol. 2017;54(3):2269‐2285. 26944285 10.1007/s12035-016-9795-4

[274] Liang Z , Zhang B , Su WW , Williams PG , Li QX . C‐Glycosylflavones alleviate tau phosphorylation and amyloid neurotoxicity through GSK3beta inhibition. ACS Chem Nerosci. 2016;7(7):912‐923. 10.1021/acschemneuro.6b00059 PMC7355085 27213824

[275] Gao C , Liu Y , Jiang Y , Ding J , Li L . Geniposide ameliorates learning memory deficits, reduces tau phosphorylation and decreases apoptosis via GSK3beta pathway in streptozotocin‐induced Alzheimer rat model. Brain Pathol. 2014;24(3):261‐269. 24329968 10.1111/bpa.12116 PMC8029432

[276] Zhang Y , Yin F , Liu J , Liu Z . Geniposide attenuates the phosphorylation of tau protein in cellular and insulin‐deficient APP/PS1 transgenic mouse model of Alzheimer's disease. Chem Biol Drug Des. 2016;87(3):409‐418. 26475430 10.1111/cbdd.12673

[277] Liu J , Liu Z , Zhang Y , Yin F . Leptin signaling plays a critical role in the geniposide‐induced decrease of tau phosphorylation. Acta Biochim Biophys Sin. 2015;47(12):1018‐1022. 26496899 10.1093/abbs/gmv106

[278] Chen Y , Wang C , Hu M , et al. Effects of ginkgolide a on okadaic acid‐induced tau hyperphosphorylation and the PI3K‐Akt signaling pathway in N2a cells. Planta Med. 2012;78(12):1337‐1341. 22700047 10.1055/s-0032-1314965

[279] Chang W , Huang D , Lo YM , et al. Protective effect of caffeic acid against Alzheimer's disease pathogenesis via modulating cerebral insulin signaling, beta‐amyloid accumulation, and synaptic plasticity in hyperinsulinemic rats. J Agric Food Chem. 2019;67(27):7684‐7693. 31203623 10.1021/acs.jafc.9b02078

[280] Ahmad Rather M , Justin‐Thenmozhi A , Manivasagam T , Saravanababu C , Guillemin GJ , Essa MM . Asiatic acid attenuated aluminum chloride‐induced tau pathology, oxidative stress and apoptosis via AKT/GSK‐3beta signaling pathway in Wistar rats. Neurotox Res. 2019;35(4):955‐968. 30671870 10.1007/s12640-019-9999-2

[281] Zhong L , Liu H , Zhang W , et al. Ellagic acid ameliorates learning and memory impairment in APP/PS1 transgenic mice via inhibition of beta‐amyloid production and tau hyperphosphorylation. Exp Ther Med. 2018;16(6):4951‐4958. 30542451 10.3892/etm.2018.6860 PMC6257515

[282] Bian Y , Chen Y , Wang X , et al. Oxyphylla A ameliorates cognitive deficits and alleviates neuropathology via the Akt‐GSK3beta and Nrf2‐Keap1‐HO‐1 pathways in vitro and in vivo murine models of Alzheimer's disease. J Adv Res. 2021;34:1‐12. 35024177 10.1016/j.jare.2021.09.002 PMC8655137

[283] Fang F , Li H , Qin T , Li M , Ma S . Thymol improves high‐fat diet‐induced cognitive deficits in mice via ameliorating brain insulin resistance and upregulating NRF2/HO‐1 pathway. Metab Brain Dis. 2017;32(2):385‐393. 27761760 10.1007/s11011-016-9921-z

[284] Li S , Zhao X , Lazarovici P , Zheng W . Artemether activation of AMPK/GSK3beta(ser9)/Nrf2 signaling confers neuroprotection towards beta‐amyloid‐induced neurotoxicity in 3xTg Alzheimer's mouse model. Oxid Med Cell Longev. 2019;2019:1862437. 31871541 10.1155/2019/1862437 PMC6907052

[285] Khan MS , Ali T , Kim MW , Jo MH , Chung JI , Kim MO . Anthocyanins improve hippocampus‐dependent memory function and prevent neurodegeneration via JNK/Akt/GSK3beta signaling in LPS‐treated adult mice. Mol Neurobiol. 2019;56(1):671‐687. 29779175 10.1007/s12035-018-1101-1

[286] Ali T , Kim T , Rehman SU , et al. Natural dietary supplementation of anthocyanins via PI3K/Akt/Nrf2/HO‐1 pathways mitigate oxidative stress, neurodegeneration, and memory impairment in a mouse model of Alzheimer's disease. Mol Neurobiol. 2018;55(7):6076‐6093. 29170981 10.1007/s12035-017-0798-6

[287] Dai S , Zhou F , Sun J , Li Y . NPD1 enhances autophagy and reduces hyperphosphorylated tau and amyloid‐beta42 by inhibiting GSK3beta activation in N2a/APP695swe cells. J Alzheimers Dis. 2021;84(2):869‐881. 34602482 10.3233/JAD-210729

[288] Zhao N , Sun C , Zheng M , Liu S , Shi R . Amentoflavone suppresses amyloid beta1‐42 neurotoxicity in Alzheimer's disease through the inhibition of pyroptosis. Life Sci. 2019;239:117043. 31722188 10.1016/j.lfs.2019.117043

[289] Rodriguez‐Urgelles E , Sancho‐Balsells A , Chen W , et al. Meridianins rescue cognitive deficits, spine density and neuroinflammation in the 5xFAD model of Alzheimer's disease. Front Pharmacol. 2022;13:791666. 35281935 10.3389/fphar.2022.791666 PMC8908099

[290] Vlassara H , Uribarri J . Advanced glycation end products (AGE) and diabetes: cause, effect, or both? Curr Diab Rep. 2014;14(1):453. 24292971 10.1007/s11892-013-0453-1 PMC3903318

[291] Zhou HH , Luo L , Zhai XD , et al. Sex‐specific neurotoxicity of dietary advanced glycation end products in APP/PS1 mice and protective roles of trehalose by inhibiting tau phosphorylation via GSK‐3beta‐TFEB. Mol Nutr Food Res. 2021;65(23):e2100464. 34669246 10.1002/mnfr.202100464

[292] An F , Zhao R , Xuan X , Xuan T , Zhang G , Wei C . Calycosin ameliorates advanced glycation end product‐induced neurodegenerative changes in cellular and rat models of diabetes‐related Alzheimer's disease. Chem Biol Interact. 2022;368:110206. 36195188 10.1016/j.cbi.2022.110206

[293] Huang L , Lin M , Zhong X , Yang H , Deng M . Galangin decreases p‐tau, Abeta42 and beta‐secretase levels, and suppresses autophagy in okadaic acid‐induced PC12 cells via an Akt/GSK3beta/mTOR signaling‐dependent mechanism. Mol Med Rep. 2019;19(3):1767‐1774. 30628698 10.3892/mmr.2019.9824

[294] Zhang N , Hu Z , Zhang Z , et al. Protective role of naringenin against Abeta(25‐35)‐caused damage via ER and PI3K/Akt‐mediated pathways. Cell Mol Neurobiol. 2018;38(2):549‐557. 28699113 10.1007/s10571-017-0519-8 PMC11482034

[295] Zhu Y , Wang J . Wogonin increases beta‐amyloid clearance and inhibits tau phosphorylation via inhibition of mammalian target of rapamycin: potential drug to treat Alzheimer's disease. Neurol Sci. 2015;36(7):1181‐1188. 25596147 10.1007/s10072-015-2070-z

[296] Song HC , Chen Y , Chen Y , et al. GSK‐3beta inhibition by curcumin mitigates amyloidogenesis via TFEB activation and anti‐oxidative activity in human neuroblastoma cells. Free Radic Res. 2020;54(11–12):918‐930. 32623920 10.1080/10715762.2020.1791843

[297] Lou H , Fan P , Perez RG , Lou H . Neuroprotective effects of linarin through activation of the PI3K/Akt pathway in amyloid‐beta‐induced neuronal cell death. Bioorg Med Chem. 2011;19(13):4021‐4027. 21652214 10.1016/j.bmc.2011.05.021

[298] Huang M , Liang Y , Chen H , Xu B , Chai C , Xing P . The role of fluoxetine in activating Wnt/beta‐catenin signaling and repressing beta‐amyloid production in an Alzheimer mouse model. Front Aging Neurosci. 2018;10:164. 29910725 10.3389/fnagi.2018.00164 PMC5992518

[299] Jin N , Zhu H , Liang X , et al. Sodium selenate activated Wnt/beta‐catenin signaling and repressed amyloid‐beta formation in a triple transgenic mouse model of Alzheimer's disease. Exp Neurol. 2017;297:36‐49. 28711506 10.1016/j.expneurol.2017.07.006

[300] Chen J , Long Z , Li Y , Luo M , Luo S , He G . Alteration of the Wnt/GSK3beta/beta‐catenin signalling pathway by rapamycin ameliorates pathology in an Alzheimer's disease model. Int J Mol Med. 2019;44(1):313‐323. 31115485 10.3892/ijmm.2019.4198

[301] Fenech RK , Hamstra SI , Finch MS , et al. Low‐dose lithium supplementation influences GSK3beta activity in a brain region specific manner in C57BL6 male mice. J Alzheimers Dis. 2023;91(2):615‐626. 36463453 10.3233/JAD-220813

[302] Jing P , Zhang JY , Ouyang Q , Wu J , Zhang XJ . Lithium treatment induces proteasomal degradation of over‐expressed acetylcholinesterase (AChE‐S) and inhibit GSK3beta. Chem Biol Interact. 2013;203(1):309‐313. 22944069 10.1016/j.cbi.2012.08.010

[303] Wu YY , Wang X , Tan L , et al. Lithium attenuates scopolamine‐induced memory deficits with inhibition of GSK‐3beta and preservation of postsynaptic components. J Alzheimers Dis. 2013;37(3):515‐527. 23948897 10.3233/JAD-130521

[304] Xiang J , Cao K , Dong YT , et al. Lithium chloride reduced the level of oxidative stress in brains and serums of APP/PS1 double transgenic mice via the regulation of GSK3beta/Nrf2/HO‐1 pathway. Int J Neurosci. 2020;130(6):564‐573. 31679397 10.1080/00207454.2019.1688808

[305] Chen S , Underwood BR , Jones PB , Lewis JR , Cardinal RN . Association between lithium use and the incidence of dementia and its subtypes: a retrospective cohort study. PLoS Med. 2022;19(3):e1003941. 35298477 10.1371/journal.pmed.1003941 PMC8929585

[306] Ali T , Kim MO . Melatonin ameliorates amyloid beta‐induced memory deficits, tau hyperphosphorylation and neurodegeneration via PI3/Akt/GSk3beta pathway in the mouse hippocampus. J Pineal Res. 2015;59(1):47‐59. 25858697 10.1111/jpi.12238

[307] Chinchalongporn V , Shukla M , Govitrapong P . Melatonin ameliorates Abeta(42)‐induced alteration of betaAPP‐processing secretases via the melatonin receptor through the Pin1/GSK3beta/NF‐kappaB pathway in SH‐SY5Y cells. J Pineal Res. 2018;64(4):e12470. 29352484 10.1111/jpi.12470

[308] Das R , Balmik AA , Chinnathambi S . Melatonin reduces GSK3beta‐mediated tau phosphorylation, enhances Nrf2 nuclear translocation and anti‐inflammation. ASN Neuro. 2020;12:1759091420981204. 33342257 10.1177/1759091420981204 PMC7754800

[309] Yao K , Zhao YF , Zu HB . Melatonin receptor stimulation by agomelatine prevents Abeta‐induced tau phosphorylation and oxidative damage in PC12 cells. Drug Des Devel Ther. 2019;13:387‐396. 10.2147/DDDT.S182684 PMC6345325 30718944

[310] Wu L , Feng X , Li T , Sun B , Khan MZ , He L . Risperidone ameliorated Abeta1‐42‐induced cognitive and hippocampal synaptic impairments in mice. Behav Brain Res. 2017;322(Pt A):145‐156. 28093254 10.1016/j.bbr.2017.01.020

[311] Ren QG , Wang YJ , Gong WG , Xu L , Zhang ZJ . Escitalopram ameliorates tau hyperphosphorylation and spatial memory deficits induced by protein kinase A activation in Sprague Dawley rats. J Alzheimers Dis. 2015;47(1):61‐71. 26402755 10.3233/JAD-143012

[312] Wu C , Gong WG , Wang YJ , et al. Escitalopram alleviates stress‐induced Alzheimer's disease‐like tau pathologies and cognitive deficits by reducing hypothalamic‐pituitary‐adrenal axis reactivity and insulin/GSK‐3beta signal pathway activity. Neurobiol Aging. 2018;67:137‐147. 29656013 10.1016/j.neurobiolaging.2018.03.011

[313] Hu JP , Xie JW , Wang CY , et al. Valproate reduces tau phosphorylation via cyclin‐dependent kinase 5 and glycogen synthase kinase 3 signaling pathways. Brain Res Bull. 2011;85(3–4):194‐200. 21435383 10.1016/j.brainresbull.2011.03.006

[314] Ubhi K , Rockenstein E , Doppler E , et al. Neurofibrillary and neurodegenerative pathology in APP‐transgenic mice injected with AAV2‐mutant TAU: neuroprotective effects of cerebrolysin. Acta Neuropathol. 2009;117(6):699‐712. 19252918 10.1007/s00401-009-0505-4 PMC3049872

[315] Zhou M , Chen S , Peng P , et al. Dulaglutide ameliorates STZ induced AD‐like impairment of learning and memory ability by modulating hyperphosphorylation of tau and NFs through GSK3beta. Biochem Biophys Res Commun. 2019;511(1):154‐160. 30773255 10.1016/j.bbrc.2019.01.103

[316] Cai HY , Holscher C , Yue XH , et al. Lixisenatide rescues spatial memory and synaptic plasticity from amyloid beta protein‐induced impairments in rats. Neuroscience. 2014;277:6‐13. 24583037 10.1016/j.neuroscience.2014.02.022

[317] Zhou D , Liu H , Li C , et al. Atorvastatin ameliorates cognitive impairment, Abeta1‐42 production and tau hyperphosphorylation in APP/PS1 transgenic mice. Metab Brain Dis. 2016;31(3):693‐703. 26883430 10.1007/s11011-016-9803-4

[318] Cuadrado‐Tejedor M , Hervias I , Ricobaraza A , et al. Sildenafil restores cognitive function without affecting beta‐amyloid burden in a mouse model of Alzheimer's disease. Br J Pharmacol. 2011;164(8):2029‐2041. 21627640 10.1111/j.1476-5381.2011.01517.x PMC3246665

[319] Shi HR , Zhu LQ , Wang SH , et al. 17beta‐estradiol attenuates glycogen synthase kinase‐3beta activation and tau hyperphosphorylation in Akt‐independent manner. J Neural Transm. 2008;115(6):879‐888. 18217188 10.1007/s00702-008-0021-z

[320] Tzeng CY , Lee WS , Liu KF , et al. Allantoin ameliorates amyloid beta‐peptide‐induced memory impairment by regulating the PI3K/Akt/GSK‐3beta signaling pathway in rats. Biomed Pharmacother. 2022;153:113389. 36076477 10.1016/j.biopha.2022.113389

[321] Ricobaraza A , Cuadrado‐Tejedor M , Perez‐Mediavilla A , Frechilla D , Del Rio J , Garcia‐Osta A . Phenylbutyrate ameliorates cognitive deficit and reduces tau pathology in an Alzheimer's disease mouse model. Neuropsychopharmacology. 2009;34(7):1721‐1732. 19145227 10.1038/npp.2008.229

[322] Abozaid OAR , Sallam MW , Ahmed ESA . Mesenchymal stem cells modulate SIRT1/MiR‐134/GSK3beta signaling pathway in a rat model of Alzheimer's disease. J Prev Alzheimers Dis. 2022;9(3):458‐468. 35841247 10.14283/jpad.2022.26

[323] Jia Y , Cao N , Zhai J , et al. HGF mediates clinical‐grade human umbilical cord‐derived mesenchymal stem cells improved functional recovery in a senescence‐accelerated mouse model of Alzheimer's disease. Adv Sci. 2020;7(17):1903809. 10.1002/advs.201903809 PMC7507104 32995116

[324] Lee IS , Jung K , Kim IS , et al. Human neural stem cells alleviate Alzheimer‐like pathology in a mouse model. Mol Neurodegener. 2015;10:38. 26293123 10.1186/s13024-015-0035-6 PMC4546205

[325] Xu AH , Yang Y , Sun YX , Zhang CD . Exogenous brain‐derived neurotrophic factor attenuates cognitive impairment induced by okadaic acid in a rat model of Alzheimer's disease. Neural Regen Res. 2018;13(12):2173‐2181. 30323150 10.4103/1673-5374.241471 PMC6199930

[326] Kazim SF , Blanchard J , Dai CL , et al. Disease modifying effect of chronic oral treatment with a neurotrophic peptidergic compound in a triple transgenic mouse model of Alzheimer's disease. Neurobiol Dis. 2014;71:110‐130. 25046994 10.1016/j.nbd.2014.07.001

[327] Wang ZJ , Han YF , Zhao F , et al. A dual GLP‐1 and Gcg receptor agonist rescues spatial memory and synaptic plasticity in APP/PS1 transgenic mice. Horm Behav. 2020;118:104640. 31765661 10.1016/j.yhbeh.2019.104640

[328] Liu S , Wang Z , Su Y , et al. A neuroanatomical basis for electroacupuncture to drive the vagal‐adrenal axis. Nature. 2021;598(7882):641‐645. 34646018 10.1038/s41586-021-04001-4 PMC9178665

[329] Xu A , Zeng Q , Tang Y , et al. Electroacupuncture protects cognition by regulating tau phosphorylation and glucose metabolism via the AKT/GSK3beta signaling pathway in Alzheimer's disease model mice. Front Neurosci. 2020;14:585476. 33328854 10.3389/fnins.2020.585476 PMC7714768

[330] Yu CC , Wang Y , Shen F , et al. High‐frequency (50 Hz) electroacupuncture ameliorates cognitive impairment in rats with amyloid beta 1‐42‐induced Alzheimer's disease. Neural Regen Res. 2018;13(10):1833‐1841. 30136700 10.4103/1673-5374.238620 PMC6128060

[331] Yu CC , Wang J , Ye SS , et al. Preventive electroacupuncture ameliorates D‐galactose‐induced Alzheimer's disease‐like pathology and memory deficits probably via inhibition of GSK3beta/mTOR signaling pathway. Evid Based Complement Alternat Med. 2020;2020:1428752. 32382276 10.1155/2020/1428752 PMC7195631

[332] Liu HL , Zhao G , Zhang H , Shi LD . Long‐term treadmill exercise inhibits the progression of Alzheimer's disease‐like neuropathology in the hippocampus of APP/PS1 transgenic mice. Behav Brain Res. 2013;256:261‐272. 23968591 10.1016/j.bbr.2013.08.008

